# The Chemical Synthesis of the Crinine and Haemanthamine Alkaloids: Biologically Active and Enantiomerically-Related Systems that Serve as Vehicles for Showcasing New Methodologies for Molecular Assembly †

**DOI:** 10.3390/molecules26030765

**Published:** 2021-02-02

**Authors:** Nan Hu, Lorenzo V. White, Ping Lan, Martin G. Banwell

**Affiliations:** Institute for Advanced and Applied Chemical Synthesis, Jinan University, Zhuhai 519070, China; hnan0423@126.com (N.H.); lorenzo.white1312@gmail.com (L.V.W.); lanpingsmzh@126.com (P.L.)

**Keywords:** alkaloid, crinine, haemanthamine

## Abstract

The title alkaloids, often referred to collectively as crinines, are a prominent group of structurally distinct natural products with additional members being reported on a regular basis. As such, and because of their often notable biological properties, they have attracted attention as synthetic targets since the mid-1950s. Such efforts continue unabated and more recent studies on these alkaloids have focused on using them as vehicles for showcasing the utility of new synthetic methods. This review provides a comprehensive survey of the nearly seventy-year history of these synthetic endeavors.

## 1. Introduction

**Figure 1:**
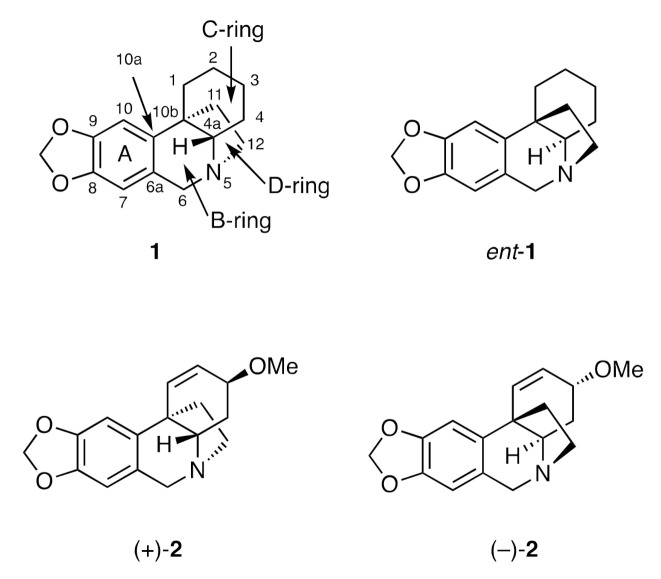
The enantiomerically related frameworks associated with the majority of the haemanthamine (**1**) and crinine (*ent-***1**) alkaloids and the structures of the (+)- and (−)- forms of buphanisine (**2**).

The alkaloids isolated from the widely distributed herbaceous and bulbous flowering plants of the amaryllis (*Amaryllidaceae*) family number more than five hundred and about 10% of these embody the 2,3,4,4a-tetrahydro-1*H*,6*H*-5,10b-ethanophenanthridine ring system (Figure 1) [1]. The 8- and 9-positions of the aromatic ring are most commonly bridged by a methylenedioxy group and depending upon whether the 5,10b-ethano-bridge is α- or β-oriented, as shown in structures **1** and *ent*-**1** respectively, the alkaloids are described as either α- or β-crinines. Those incorporating the former framework are sometimes denoted as haemanthamine alkaloids while those embodying the enantiomerically related one are described as crinines So, for example, (+)-buphanisine [(+)-**2**] (isolated from the widely distributed plant *Sternbergia sicula*) [2] and (–)-buphanisine [(–)-**2**] (isolated from the Central African plant *Boöphane fischeri*) [3] are described as α- and β-crinines, respectively. These days it is common for compounds in either enantiomeric series to be described, collectively, as crinines [1].

**Figure 2:**
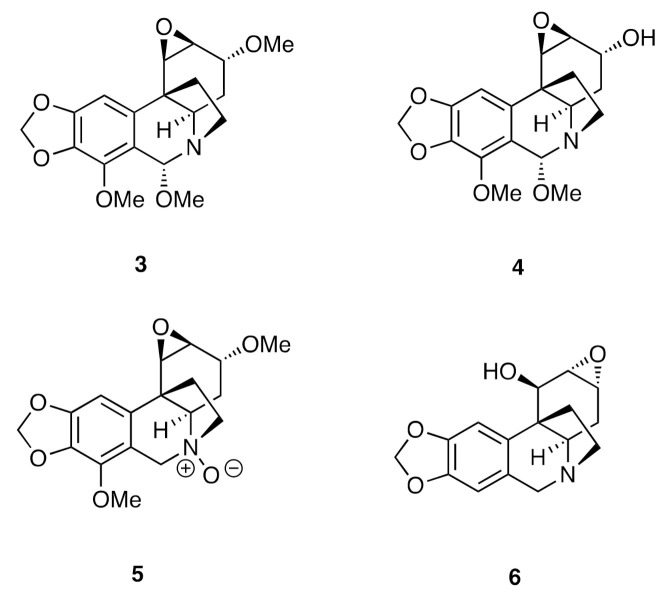
Examples of novel crinine alkaloid structures reported recently.

New members of these families continue to be identified on a regular basis with the alkaloids **3**–**6** (Figure 2) having been reported recently. 

The first three of these newer natural products were isolated from *Crinum latifolium* collected in Hanoi [4] and the fourth from *Crinum jagus* found in Senegal [5]. Their structures highlight the functional and stereochemical diversity that can be encountered within this family of alkaloids [1].

**Scheme 1:**
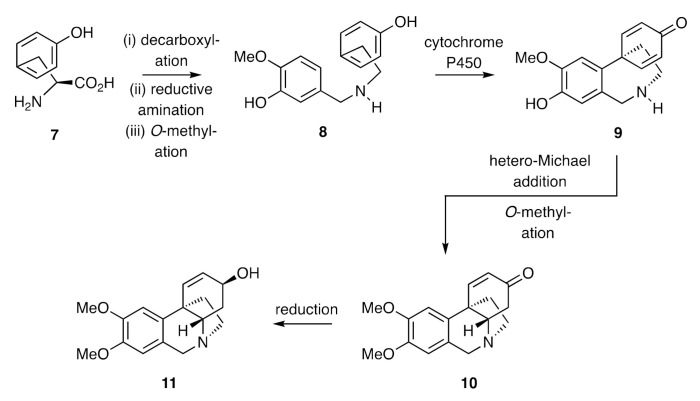
The biosynthetic pathway leading from *L*-tyrosine (**7**) to (+)-maritidine (**11**).

The crinines are thought to arise in vivo by the pathway shown in Scheme 1 and wherein *L*-tyrosine (**7**) is first converted into *O*-methylnorbelladine (**8**) that itself undergoes intramolecular oxidative *para*-*para* phenolic coupling to afford the cyclohexadienone **9** [6,7]. The nitrogen associated with this last compound is perfectly poised to add, via an enzymatically-controlled hetero-Michael addition process, to the prochiral cyclohexadienone moiety (of **9**), thereby affording, depending upon the enantiomeric form of the final product, either the tetracyclic compound **10** or its enantiomer. In the case of the illustrated sequence, the carbonyl group within enone **10** is then reduced diastereoselectively to afford (+)-maritidine (**11**), this being a rare example of an haemanthamine/crinine-type alkaloid that has methoxy groups rather than a methylenedioxy group attached to the aromatic ring.

While none of compounds **3**–**6**, for example, show any notable biological activities in the limited assays used to evaluate them thus far [4,5], collectively speaking, the crinine alkaloids display a remarkable range of often-pronounced biological effects. These include anti-viral, anti-bacterial, anti-proliferative, anti-malarial, anti-plasmodial, and apoptosis-inducing properties [1,8,9,10,11,12,13]. Furthermore, analoguing and derivatization studies have provided compounds that show an extended range of activities [14,15,16,17,18,19].

The distinctive structural features of the crinine and haemanthamine alkaloids together with their remarkable range of biological activities have prompted extensive efforts, over many decades, to develop synthetic routes to them. Indeed, more recently, they have become popular vehicles for showcasing the utility of newly developed synthetic methods. This review seeks to provide, as its primary focus, a comprehensive survey of the many means by which these natural products, or at least the associated frameworks, can be assembled. No coverage of the literature concerning the synthesis of the C3a-arylated perhydroindole-containing mesembrine alkaloids [20], natural products which embody the A-, C- and D-rings of the crinines, is provided, except where such efforts are directly related to accessing the title natural products.

### 1.1. The Chemical Synthesis of Crinine Alkaloids

In seeking to provide both a comprehensive and logical survey of the large body of work describing syntheses of or synthetic approaches to the title alkaloids [21], the studies described herein have been categorized according to the final bond-forming event used in the assembly of the associated ABCD (tetracyclic) ring system, and these are presented in alphabetical order (viz. closing stage B-ring formation sequences are presented before BD-ring forming processes that are presented before C-ring formation processes, etc). As revealed below, only five different approaches have been reported thus far with two of these dominating. Of course, it follows that numerous other approaches are possible, and so it would appear that many further opportunities exist for developing synthetic protocols that afford these alkaloids.

In all of these endeavors, an inevitable and key concern is the assembly of the all-carbon quaternary centre C10b. The manner in which this challenge is addressed within each synthesis is provided as part of the discussions presented below. Within three of the five different approaches mentioned in the previous paragraph, optically active products are obtained in a number of instances, particularly among more recent reports. However, these have not been presented in separate sub-sections but, rather, intermingled with approaches that produce racemic materials, this being done in an effort to group together ones involving related strategies. Where reaction sequences lead to racemic products, then the (±) prefix has been applied to both the target compound numbers as well as to those of the precurors and intermediates that incorporate stereogenic centers. Conversely, in those synthetic sequences providing enantiomerically enriched (or pure) target compounds, either the (+) and/or (−) prefixes are used, except in those instances where the specific rotation of the intermediates or precursors have not been reported or were not readily available. In such cases, and where it can be logically assumed that such compounds are optically active, a compound number lacking a prefix is used. By such means, it is hoped that the reader can discern, at a glance, whether the synthesis being described in a given scheme leads to racemic material or is, instead, asymmetric.

(a) B-Ring Formation via Creation of the C6-C6a Bond (Pictet-Spengler Cyclization)

**Scheme 2:**
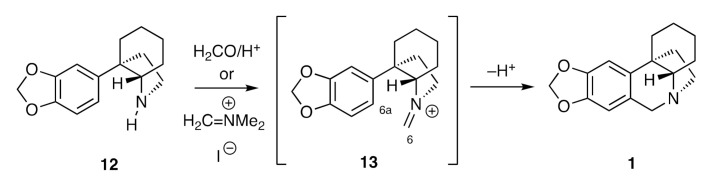
C6–C6a bond formation via Pictet–Spengler cyclisation leading to B-ring assembly and completion of the synthesis of the target ABCD-ring system **1**.

By far the most prevalent means of completing the assembly of 2,3,4,4a-tetrahydro-1*H*,6*H*-5,10b-ethanophenanthridine ring system (**1**) associated with the crinine alkaloids is via the pathway shown, in generalized terms, in Scheme 2. To such ends, a C3a-arylated perhydroindole such as compound **12** is treated with either formaldehyde and a protic acid or a species such as Eschenmoser’s salt (H_2_C=NMe_2_^+^I^−^). The enusing imminium ion **13** then engages in a Pictet–Spengler cyclization reaction to afford the target framework **1**.

**Scheme 3:**
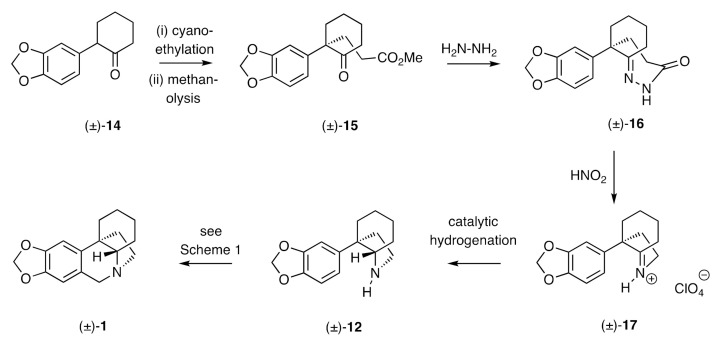
The original (Wildman) synthesis of (±)-crinane [(±)-**1**] (detailed reaction conditions and yields were not provided in the original publication).

This end-game comprised the key step of the very first synthesis of the crinane framework reported by Wildman in 1956 [22]. Thus, as shown in Scheme 3, the α-arylated cyclohexanone (±)-**14** was converted, over two steps, into the keto-ester (±)-**15**. Upon reaction with hydrazine, the latter compound afforded a hydrazine hydrazide presumed (by the present authors) to be compound (±)-**16**, which, upon exposure to nitrous acid, gave the cyclic imine (±)-**17**. Catalytic hydrogenation of this last species followed by a Pictet–Spengler reaction then gave, presumably via the octahydroindole (±)-**12**, the basic ring system (±)-**1**. Interestingly, compound (±)-**1**, which is not, in either enantiomeric form, a natural product, displays analgesic properties [22].

**Scheme 4:**
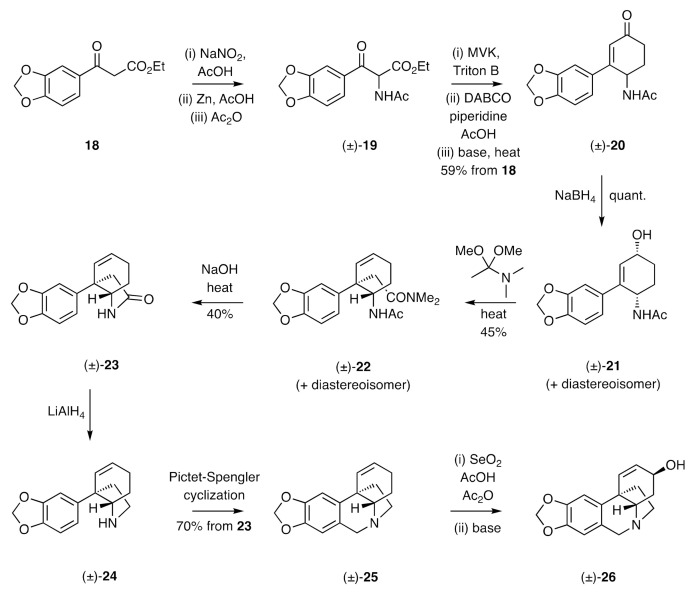
Muxfeldt’s total synthesis of (±)-crinine [(±)-**26**].

The first total synthesis of a crinine alkaloid was reported by Muxfeldt in 1966 [23]. As shown in Scheme 4, β-keto-ester **18** was reacted with in situ-generated nitrous acid to yield an α-nitroso-derivative that was reduced with zinc in acetic acid. Acetylation of the resulting amine then gave acetamide (±)-**19** and Michael addition of the last compound to methyl vinyl ketone (MVK), cyclization of the resulting adduct followed by saponification of the carbomethoxy group and decarboxylation of the resulting acid, then gave compound (±)-**20**. Upon reduction with sodium borohydride, this compound was converted into allylic alcohol (±)-**21** and its corresponding *trans*-isomer. Heating this mixture with dimethylacetamide dimethyl acetal in either refluxing benzene or toluene resulted in an Eschenmoser–Claisen rearrangement, thereby affording a mixture of compound (±)-**22** and its diastereoisomer in 45% combined yield. This mixture was accompanied by the cyclohexa-1,3-diene resulting from dehydration of substrate (±)-**21** and/or its diastereoisomer. Saponification of the mixture of compound (±)-**22** and its diastereoisomer using sodium hydroxide afforded lactam (±)-**23** that was converted into the C3a-arylated hexahydroindole (±)-**24** on treatment with lithium aluminium hydride. Subjection of this last compound to a Pictet–Spengler reaction (precise conditions not given) afforded the 5,10b-ethanophenanthridine (±)-**25**, which was converted, through a SeO_2_-mediated allylic oxidation reaction, into (±)-crinine [(±)-**26**]. A notable feature of this synthesis was the creation of the quaternary carbon center of the final target by subjecting a C3-arylated cyclohex-2-en-1-ol to a Claisen-type reaction. This strategy has, as detailed below, been used in several other studies.

**Scheme 5:**
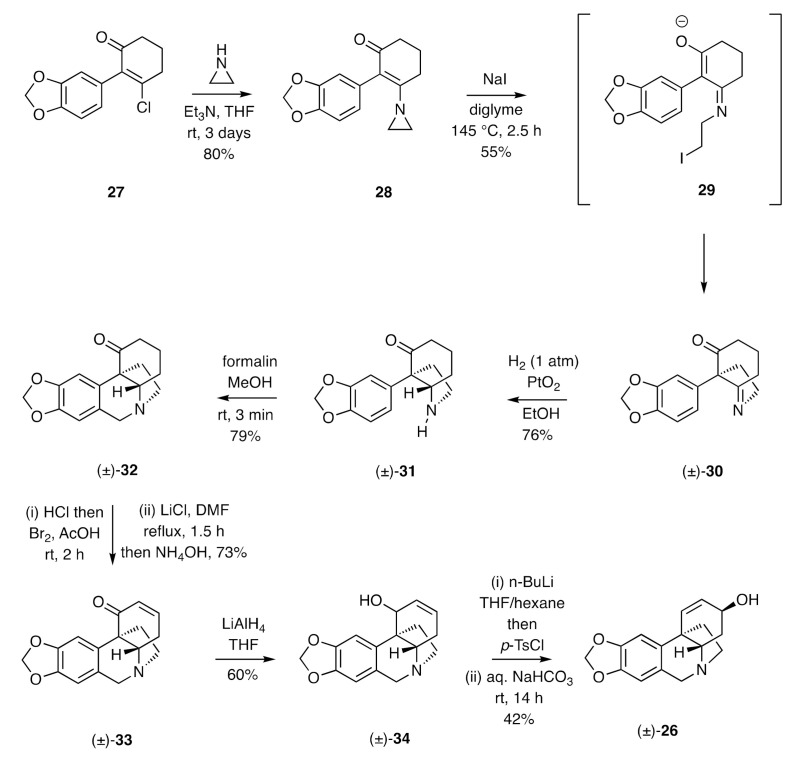
Whitlock’s total synthesis of (±)-crinine [(±)-**26**].

In 1967, Whitlock described [24,25] a synthesis of (±)-crinine (Scheme 5). This involved, in its early stages, reaction of the α-arylated β-chloroenone **27** with aziridine to afford product **28** that upon treatment with sodium iodide at elevated temperatures gave, presumably through cyclization of intermediate **29**, the hexahydroindole (±)-**30** incorporating the quaternary carbon of the target. Catalytic hydrogenation of this last compound afforded a mixture of the *cis*-configured octahydroindole (±)-**31** (76%) and the corresponding *trans*-isomer (5.5%). Subjection of the former product to a Pictet–Spengler reaction with formalin in methanol gave compound (±)-**32**. This was then submitted to an α-bromination/dehydrobromination sequence to afford the enone (±)-**33** (73%), which was itself reduced with lithium aluminium hydride to the corresponding allylic alcohol, (±)-**34** seemingly as a single diastereoisomer but of undefined configuration. Tosylation of compound (±)-**34** and hydrolysis of the resulting ester in aqueous sodium bicarbonate then afforded (±)-crinine [(±)-**26**] in 42% yield.

**Scheme 6:**
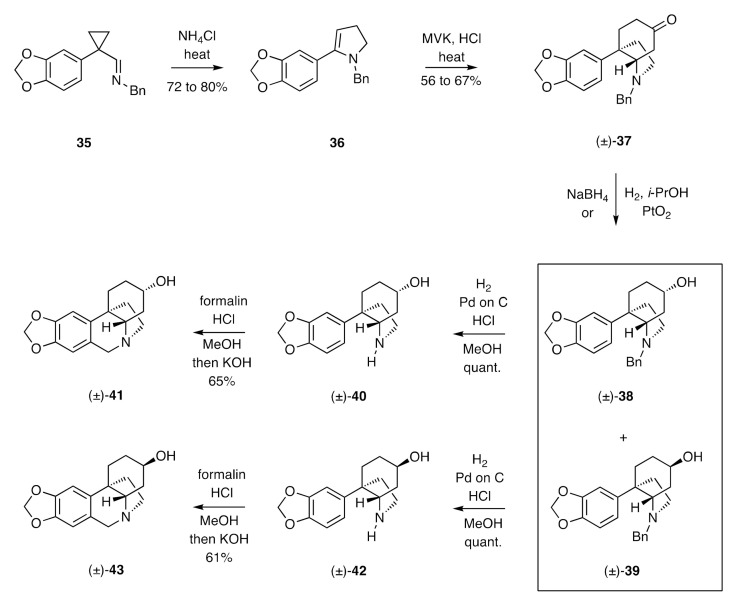
Stevens’ total syntheses of (±)-3-*epi*-elwesine [(±)-**41**] and (±)-elwesine [(±)-**43**].

Stevens’ syntheses of (±)-elwesine and its C3-epimer (Scheme 6), reported in the early 1970s [26,27], provided another elegant example of the synthesis of a C3a-arylated perhydroindole that then served as the substrate for a concluding Pictet–Spengler cyclisation reaction. Thus, the iminocyclopropane **35**, obtained from piperonyl cyanide, was subjected to thermal rearrangement in the presence of ammonium chloride to give the isomeric dihydropyridine **36**. This compound was engaged in an annulation reaction with methyl vinyl ketone (MVK), thus allowing for assembly of the crinine C-ring and thereby the C3a-arylperhydroindole (±)-**37**. Reduction of this last compound with sodium borohydride afforded a 3:1 mixture of the epimeric alcohols (±)-**38** and (±)-**39** (no yield specified), while reduction with hydrogen and PtO_2_ in *i*-propanol afforded a 1:8 mixture of the same products in 96% combined yield. Hydrogenolytic cleavage of the benzyl group attached at nitrogen in compound (±)-**38** was achieved using hydrogen and 10% Pd on C. The product 2°-amine (±)-**40** was then subjected to standard Pictet–Spengler conditions to afford (±)-3-*epi*-elwesine [(±)-**41**]. In an analogous sequence of reactions, compound (±)-**39** was converted, via amine (±)-**42**, into (±)-elwesine [(±)-**43**].

**Scheme 7:**
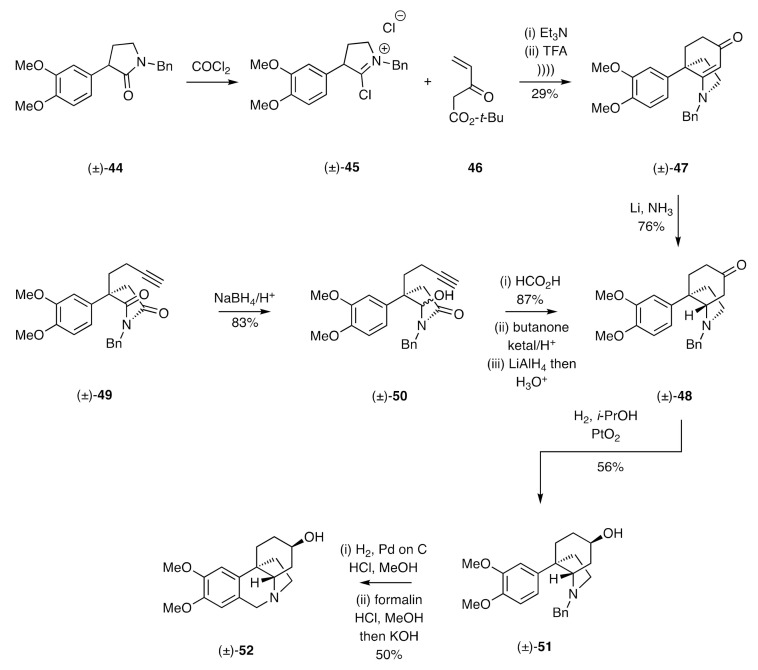
The Michael and Speckamp syntheses of (±)-dihydromaritidine [(±)-**52**].

A C-ring annulation protocol related to the one described immediately above was employed by Michael in a formal total synthesis of (±)-dihydromaritidine [28]. Thus, as shown in the early parts of Scheme 7, treatment of the *N*-benzylated lactam (±)-**44** with phosgene afforded the *C*-chloroiminium ion (±)-**45** that was reacted with the MVK derivative **46** in the presence of triethylamine. The primary condensation products of this process were then reacted with trifluoroacetic acid (TFA) under ultrasonic irradiation conditions to afford cyclohexenone (±)-**47**, albeit in only 29% yield. Subjection of this last compound to reduction with lithium in liquid ammonia then gave the saturated equivalent (±)-**48** stereoselectively. Compound (±)-**48** was a key intermediate associated with the Speckamp [29] total synthesis (±)-dihydromaritidine that involved, as a key step, the regioselective reduction of imide (±)-**49** to the carbinol-lactam (±)-**50**. Exposure of this last compound to formic acid gave, via cyclization of an in situ-generated α-acylimmonium ion with the pendant alkyne, the expected cyclohexanone annulated lactam. Selective protection of the associated ketone residue as the corresponding ethylene ketal, reductive cleavage of the lactam carbonyl, and deprotection of the cyclohexanone then provided compound (±)-**48**, which was selectively reduced under the conditions established by Stevens (*cf* (±)-**37** → (±)-**39** in Scheme 6), thus affording alcohol (±)-**51**. Carrying this compound forward, also under conditions established by Stevens, gave (±)-dihydromaritidine [(±)-**52**].

**Scheme 8:**
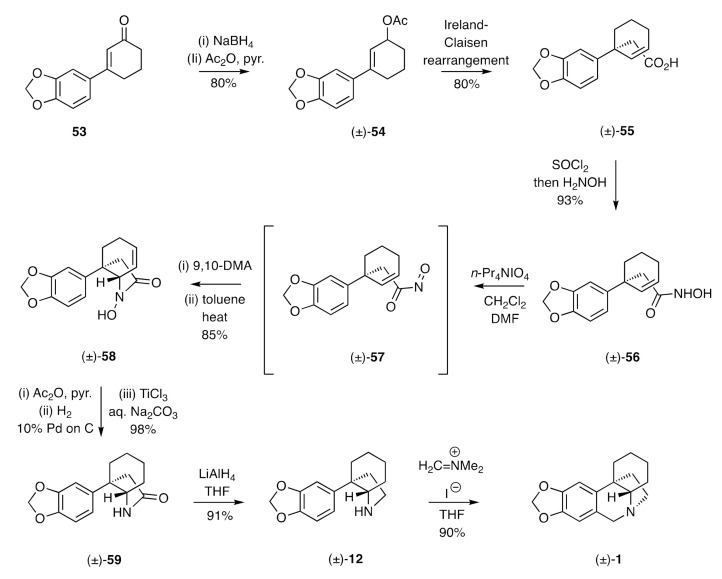
The Keck synthesis of (±)-crinane [(±)-**1**].

The Keck synthesis of (±)-crinane [(±)-**1**] [30,31] involved, as shown in Scheme 8, initial 1,2-reduction of the β-arylated cyclohexenone **53** and acetylation of the ensuing allylic alcohol to give ester (±)-**54**. This last compound was subject to an Ireland–Claisen rearrangement reaction, and the product acid (±)-**55** thus obtained was converted into the corresponding hydroxamic acid (±)-**56** under standard conditions. Compound (±)-**56** was oxidized to its acylnitroso derivative (±)-**57** using tetrapropylammonium periodate and then trapped in a Diels–Alder reaction with 9,10-dimethylanthracene (9,10-DMA). On heating the isolable and crystalline adduct of this reaction in refluxing toluene for 0.25 h, a cycloreversion reaction took place to regenerate compound (±)-**57** that underwent, in situ, an intramolecular ene reaction, thus delivering the *N*-hydroxylactam (±)-**58** embodying the ACD-ring system of the crinines. This “catch and release” protocol was necessary, because cyclic hydroxamic acids, of which (±)-**58** is an example, are known to be unstable under oxidizing conditions (being converted into reactive acyl nitroxyl species). Conversion of compound (±)-**58** into the corresponding acetate, hydrogenation of the cyclohexene double bond, and reductive cleavage of the acetate group using TiCl_3_ then gave lactam (±)-**59**, which was reduced to the cyclic amine (±)-**12** using LiAlH_4_. While this last compound is an established precursor to crinane [(±)-**1**] (see Scheme 2), Keck and Webb showed that the necessary Pictet–Spengler cyclisation was best accomplished using Eschenmoser’s salt rather than, for example, formaldehyde/HCl (95% vs 53% yield). Adaptations of this reaction sequence have provided a synthesis of (±)-dihydromaritidine [(±)-**52**].

**Scheme 9:**
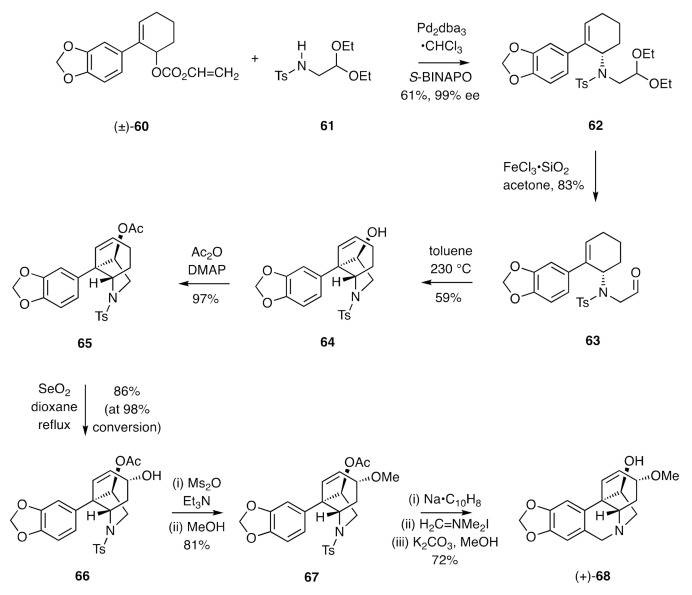
The Mori synthesis of (+)-crinamine [(+)-**68**].

In more recent work, Mori and co-workers [32] have employed a thermally-induced carbonyl-ene reaction as a key step in developing an asymmetric total synthesis of (+)-crinamine (Scheme 9). Thus, the racemic allylic carbonate (±)-**60** was subjected to reaction with the sulfonamide **61** in the presence of a palladium catalyst and the homochiral ligand *S*-BINAPO to afford the allylic sulfonamide **62**, which was obtained in 99% ee. Cleavage of the associated acetal residue using ferric chloride on silica in acetone gave the aldehyde **63** that engaged in an intramolecular carbonyl ene reaction on heating at 230 °C in toluene. The resulting tricyclic alcohol **64** was acetylated under standard conditions to give the ester **65**. Alyllic oxidation of the last compound using selenium dioxide afforded compound **66**, and the mesylate derived from this was subjected to methanolysis, a process that proceeded largely with retention of configuration and thus providing methyl ether **67**. Exposure of compound **67** to sodium naphthalenide resulted in cleavage of the tosyl group, and the amine so-revealed was treated with Eschenmoser’s reagent. The ensuing Pictet–Spengler product was treated with methanolic potassium carbonate to afford (+)-crinamine [(+)-**68**].

**Figure 3:**
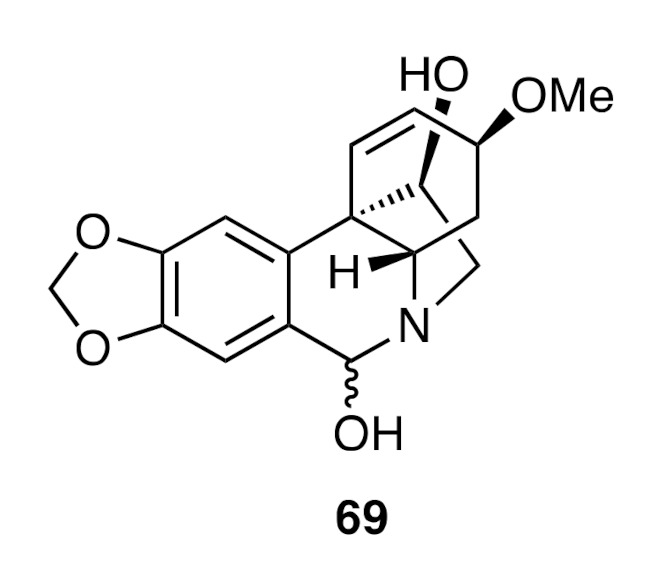
The structure of (−)-haemanthidine (**69**).

A related sequence of reactions starting from allylic alcohol **66** provided (−)-haemanthidine (**69**) (Figure 3).

**Scheme 10:**
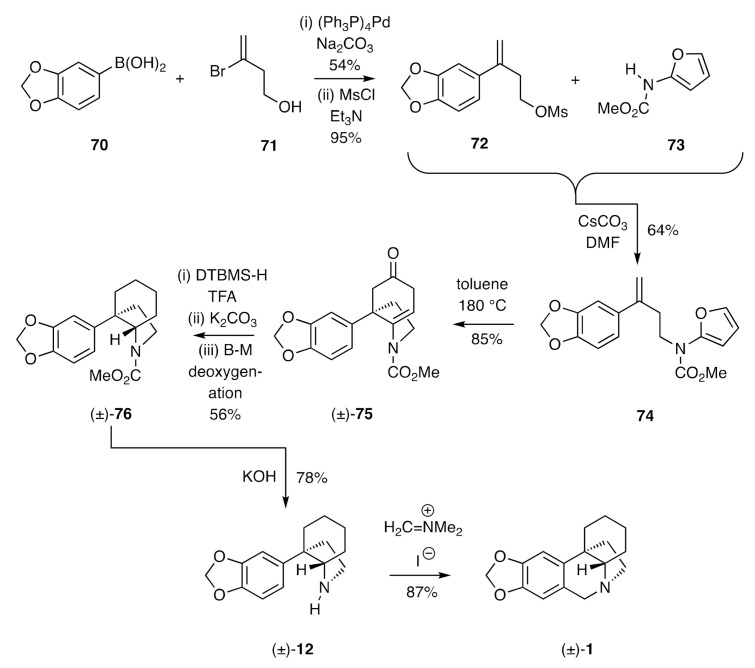
The Padwa synthesis of (±)-crinane [(±)-**1**].

In another notable demonstration of the capacity of pericyclic processes to establish the crinane framework, Padwa [33] has described a synthesis of compound (±)-**1** using an intramolecular Diels-Alder (IMDA) reaction of furan-tethered styrene as the key step. So, as shown in Scheme 10, Suzuki–Miyaura cross-coupling of arylboronic acid **70** with bromoalkene **71** afforded the corresponding styrene alcohol that could be converted into the mesylate **72** under standard conditions. Reaction of this ester with the furanyl carbamate **73** in the presence of caesium carbonate then gave the furanylated styrene **74**. Heating a toluene solution of this last compound at 180 °C in a sealed tube resulted in the anticipated IMDA reaction taking place with the initially formed adduct itself fragmenting to give the observed β,γ-unsaturated cyclohexanone (±)-**75**. Ionic hydrogenation of this last compound using di-*t*-butylmethylsilane (DTBMS-H) and TFA resulted in reduction of the C=C bond and the ketone carbonyl to give, after potassium carbonate-mediated cleavage of the intermediate and epimeric trifluoroacetates, a mixture of alcohols. These were then subjected to a Barton–McCombie (B–M) deoxygenation process and thus the affording compound (±)-**76**. Cleavage of the carbamate residue associated with the last compound was achieved using KOH, and the amine (±)-**12** so-formed was converted into (±)-crinane [(±)-**1**] using Eschenmoser’s salt.

**Scheme 11:**
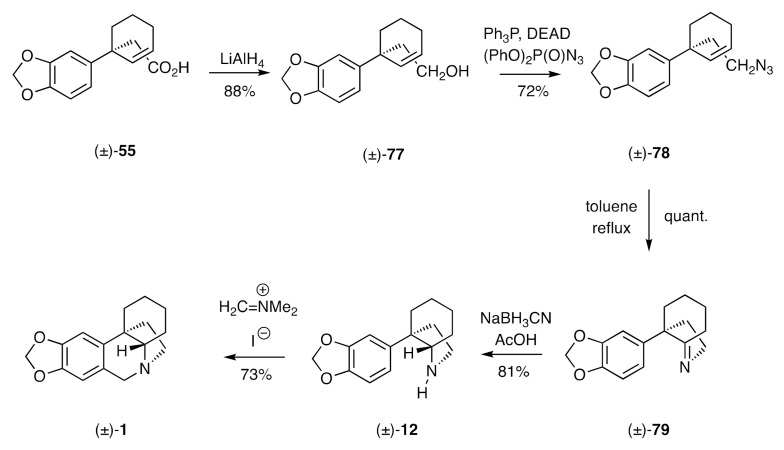
The Pearson synthesis of (±)-crinane [(±)-**1**].

An intramolecular [3+2] azide-olefin cycloaddition reaction has been employed by Pearson [34] as the key step in yet another synthesis of (±)-crinane [(±)-**1**]. In this work, the carboxylic acid (±)-**55** associated with the Keck synthesis (Scheme 8) was reduced (Scheme 11) to the corresponding alcohol (±)-**77** using LiAlH_4_. This alcohol was engaged in a Mitsunobu reaction to afford the unsaturated azide (±)-**78**. Simply heating this last compound in refluxing toluene promoted the anticipated addition reaction, thus affording, after nitrogen extrusion from the primary reaction product, the cyclic imine (±)-**79**. Diastereoselective reduction of this last compound gave the by now familiar C3a-arylperhydroindole (±)-**12**, which was readily converted, by the usual means, into (±)-crinane [(±)-**1**].

**Scheme 12:**
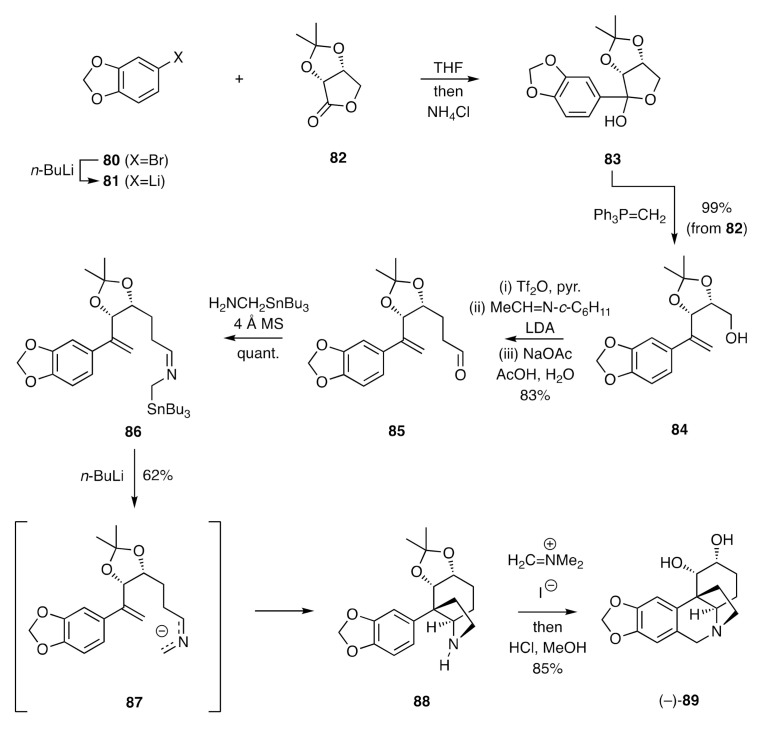
The Pearson synthesis of (−)-amabiline [(−)-**89**].

A related strategy was employed for assembling the ACD-ring system of the dihydroxylated crinine alkaloid (−)-amabiline [35,36,37]. This total synthesis started with the conversion (Scheme 12) of arylbromide **80** into the lithio-species **81** and its addition to the homochiral lactone **82**. The resulting lactol **83** was subjected to a Wittig olefin reaction, and the product styrene-alcohol **84** converted into the corresponding triflate, which was itself reacted with the anion derived from deprotonation of *N*-(cyclohexyl)acetaldimine. Hydrolysis of the product so-formed under mildly acidic conditions then gave aldehyde **85**. A Schiff base condensation between this last compound and (aminomethyl)tri-*n*-butylstannane then gave the crucial (2-azaallyl)stannane **86**. Upon treatment of compound **86** with *n*-BuLi, this (presumably) formed the corresponding 2-azaallyl anion **87** that engaged in an intramolecular and diastereofacially selective [π4s + π2s] cycloadditon with the pendant styrene double-bond to afford adduct **88**. This was readily converted, by sequential treatment with Eschenmoser’s salt and then methanolic HCl, into (–)-amabiline (**89**).

In an adaptation of the chemistry used by Pearson for the synthesis of (±)-crinane (Scheme 11) and the early stages of the Keck synthesis of the same (Scheme 8), Akai has used a combination of an oxovanadium complex and a lipase to effect the dynamic kinetic resolution of allylic acetate (±)-**54 [38]** and thereby establish a total synthesis of (−)-crinane [(−)-**1**].

**Scheme 13:**
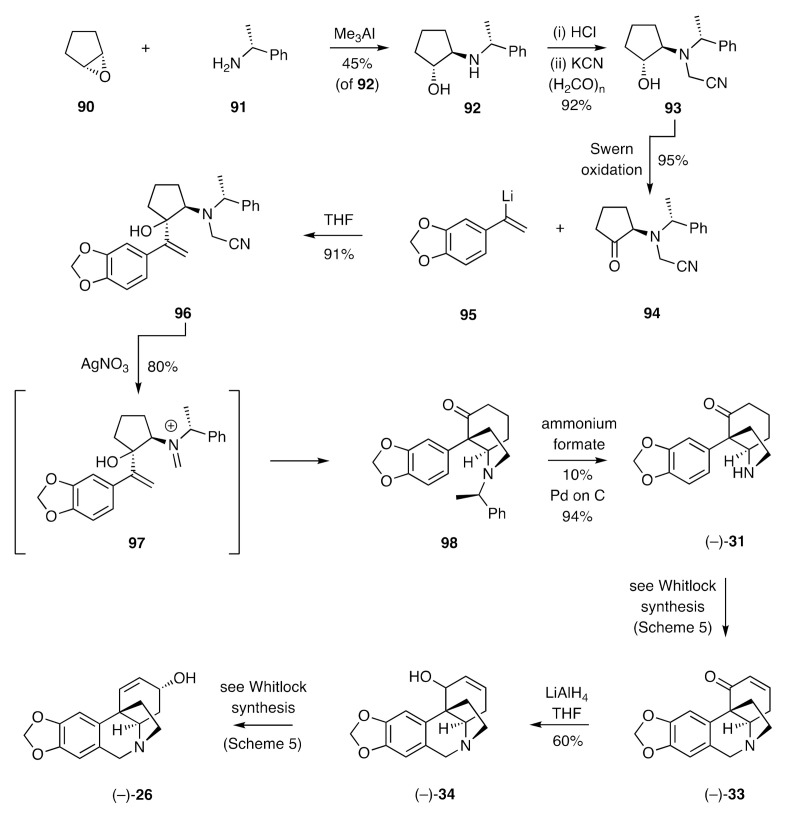
The Overman synthesis of (−)-crinine [(−)-**26**].

A cationic aza-Cope rearrangement/Mannich cyclization sequence served as the pivotal process employed by Overman [39,40] in establishing a synthesis of (−)-crinine (Scheme 13). Thus, cyclopentene oxide (**90**) was reacted with the α-chiral 1°-amine **91** in the presence of trimethylaluminium to give a 1:1 mixture of amino-alcohol **92** (45%) and its diastereoisomer. These isomers were readily separated by conventional chromatographic methods, and the hydrochloride salt of compound **92** was treated with potassium cyanide then paraformaldehyde to give the α-aminonitrile **93**. Swern oxidation of this last compound then gave the ketone **94**, which was itself reacted in a diastereoselective manner with the styryl lithium **95** to give the key substrate **96**. Treatment of compound **96** with silver nitrate then gave, presumably via the initially formed iminium ion **97** that engages in an aza-Cope rearrangement/Mannich cyclization, compound **98**. The chiral auxiliary associated with the last compound was cleaved using transfer hydrogenolysis techniques to give ketone (−)-**31**, the racemic form of which served as a late-stage intermediate in the Whitlock synthesis of (±)-crinine. Accordingly, the homochiral material was carried forward in the same manner to give, via enone (−)-**33** and allylic alcohol (−)-**34**, (−)-crinine [(−)-**26**].

**Scheme 14:**
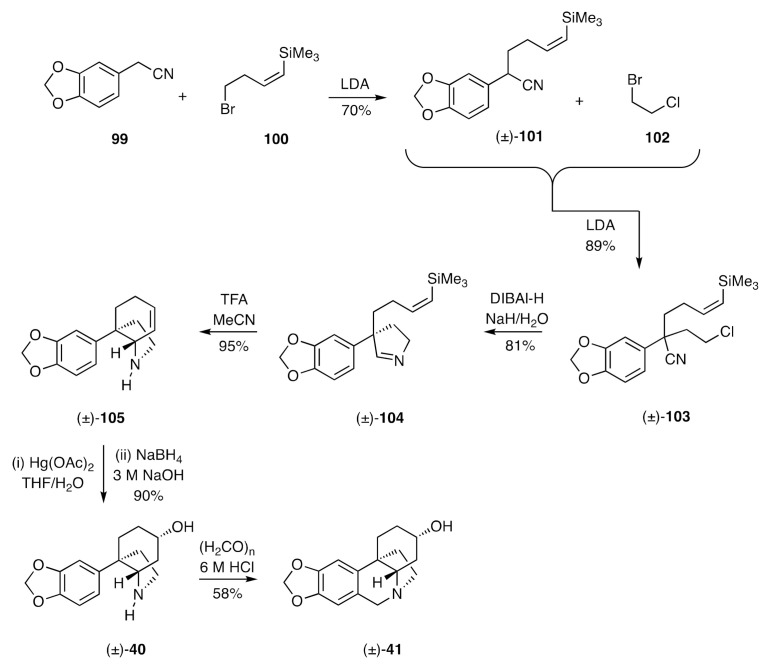
Overman’s total synthesis of (±)-3-*epi*-elwesine [(±)-**41**].

A distinctly different means of forming the CD-ring system of the crinines was employed by Overman in establishing a synthesis of (±)-3-*epi*-elwesine [41,42]. As shown in Scheme 14, the arylated acetonitrile **99** was alkylated using the bromide **100** in the presence of lithium diethylamide (LDA) to give product (±)-**101**, which was itself subjected to alkylation using bromide **102** as the electrophile. The nitrile group associated with the product of this process, (±)-**103**, was then reduced with *i*-Bu_2_AlH (DIBAl-H), and the resulting *N*-metallated imine reacted, in situ, with the tethered alkyl chloride to give the cyclic imine (±)-**104**. On treatment of the last compound with TFA, the derived iminium ion reacted, in a cationic cyclization process, with the pendant, *Z*-configured alkenylsilane to give the tricyclic product (±)-**105** (this conversion is reminiscent of the conversion (±)-**50** → (±)-**48** employed in the Speckamp synthesis of (±)-dihydromaritidine [(±)-**52**]—see Scheme 7}). Oxymercuration of the cyclohexenyl double bond within compound (±)-**105** proceeded both regio- and diastereo-selectively to give, after demercuration, alcohol (±)-**40**, an advanced intermediate associated with Steven’s synthesis of (±)-3-*epi*-elwesine [(±)-**41**] (Scheme 6). Reaction of compound (±)-**40** under Pictet–Spengler conditions then gave (±)-3-*epi*-elwesine [(±)-**41**].

**Scheme 15:**
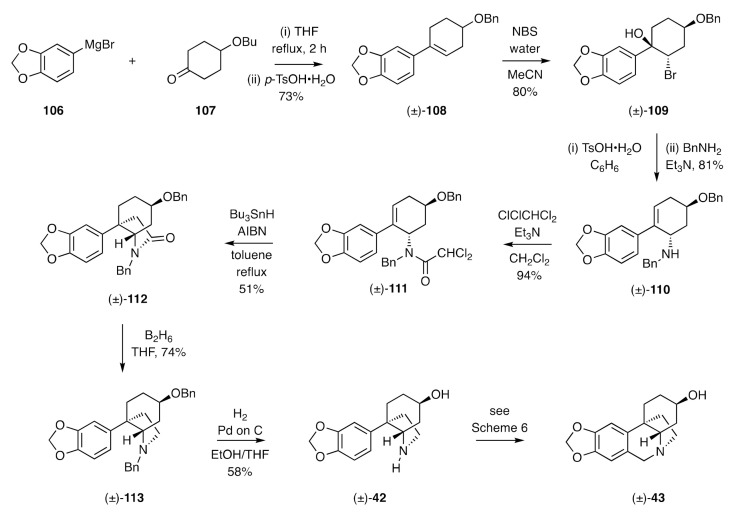
Ishibashi’s total synthesis of (±)-elwesine [(±)-**43**].

Radical cyclization reactions have also featured as key steps in the assembly of the CD-ring system of the crinane alkaloids. So, for example, in 1991, Ishibashi reported [43] a synthesis of (±)-elwesine [(±)-**43**] (Scheme 15) that commenced with the addition of the aryl Grignard **106** to the 4-substituted cyclohexanone **107** and dehydration of the resulting 3°-alcohol to give styrene (±)-**108**. Treatment of this last compound with *N*-bromosuccinimide (NBS) in wet acetonitrile gave, as the major product of reaction, bromohydrin (±)-**109**, and this compound was subjected to an acid-catalyzed dehydration reaction, and the ensuing allylic bromide then reacted with benzylamine to afford the 3°-amine (±)-**110** as the major product of reaction. Acylation of compound (±)-**110** with dichloroacetyl chloride then gave amide (±)-**111** that, upon reaction with tri-*n*-butylstannane in the presence of the radical initiator azobisisobutyronitrile (AIBN), engaged in a 5-*exo*-*trig* radical cyclization reaction and then (presumably) a subsequent radical dechlorination reaction to give lactam (±)-**112** in modest yield. Reductive cleavage of the carbonyl unit associated with the last compound was achieved using diborane, and the two benzyl groups in product (±)-**113** were cleaved under hydrogenolytic conditions to afford the amino-alcohol (±)-**42**, the immediate precursor to (±)-elwesine [(±)-**43**] associated with the Steven’s synthesis (Scheme 6) of the same target.

**Figure 4:**
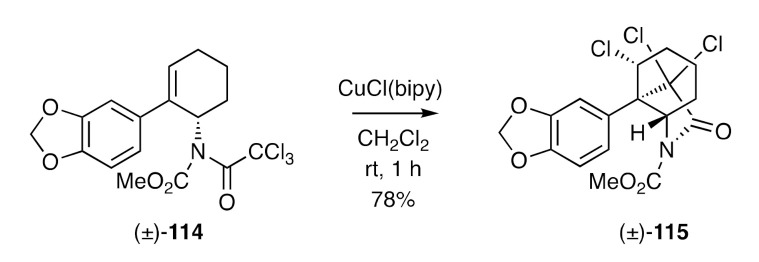
The key step associated with Nagashima’s formal synthesis of (±)-crinane [(±)-**1**].

A closely related approach to crinane has been reported [44] by Nagashima et al. who employed, as the key step (Figure 4), a Cu(I)-catalyzed atom-transfer reaction of trichloroacetamide (±)-**114** that afforded the isomeric lactam (±)-**115**. This process was presumed to proceed via a radical cyclization pathway. 

**Scheme 16:**
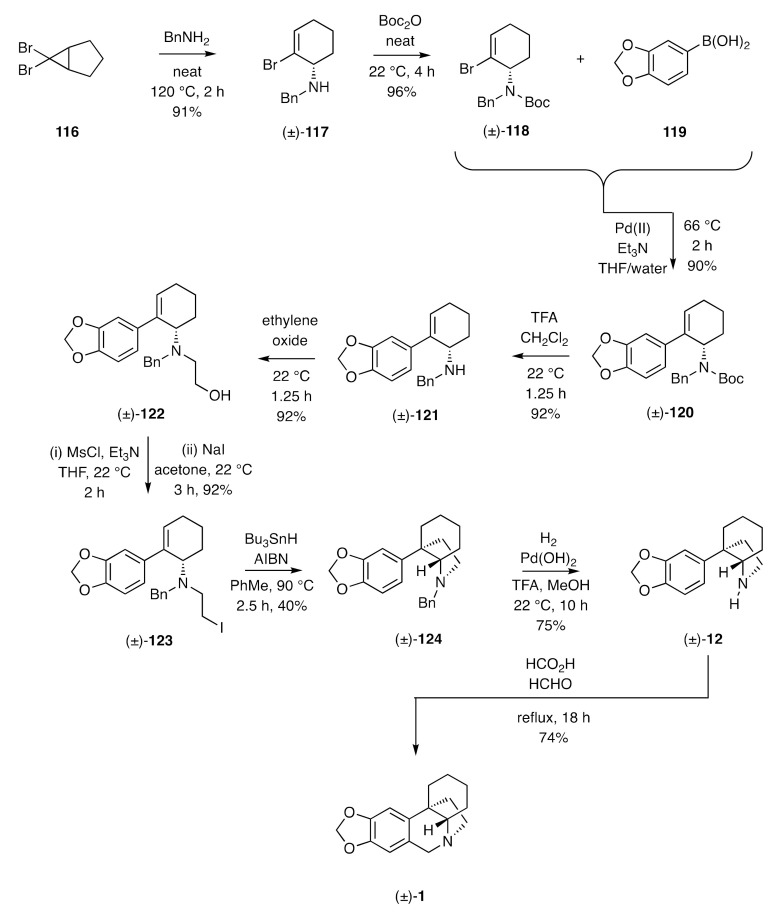
Lan’s radical cyclization route to (±)-crinane [(±)-**1**].

In our own research, we have established an alternative 5-*exo*-*trig*-based radical cyclization pathway to (±)-crinane [(±)-**1**]. This starts (Scheme 16) [45] with the thermally-induced electrocyclic ring-opening of the ring-fused *gem*-dibromocyclopropane **116** and trapping of the resulting ring-expanded π-allyl cation by benzylamine and so affording the allylic amine (±)-**117**. Conversion of the latter compound, under standard conditions, into the Boc-protected derivative (±)-**118** followed by cross-coupling of this with aryl boronic acid **119** under Suzuki-Miyaura conditions then afforded the anticipated product (±)-**120**. Treatment of this intermediate with TFA resulted in Boc-group cleavage to deliver the 2°-amine (±)-**121**. *N*-Alkylation of compound (±)-**121** with ethylene oxide afforded the amino-alcohol (±)-**122**, which was mesylated in situ and the resulting ester subjected to a Finkelstein reaction to give iodide (±)-**123**. In the pivotal step of the reaction sequence, this halide was treated with Bu_3_SnH and AIBN in hot toluene to promote a 5-*exo-trig* radical cyclization and thereby generate the C3a-arylperhydroindole (±)-**124** albeit rather inefficiently. Hydrogenolytic cleavage of the benzyl residue associated with this product afforded compound (±)-**12** that was readily converted into (±)-crinane [(±)-**1**] on treatment with formaldehyde in the presence of formic acid. 

**Scheme 17:**
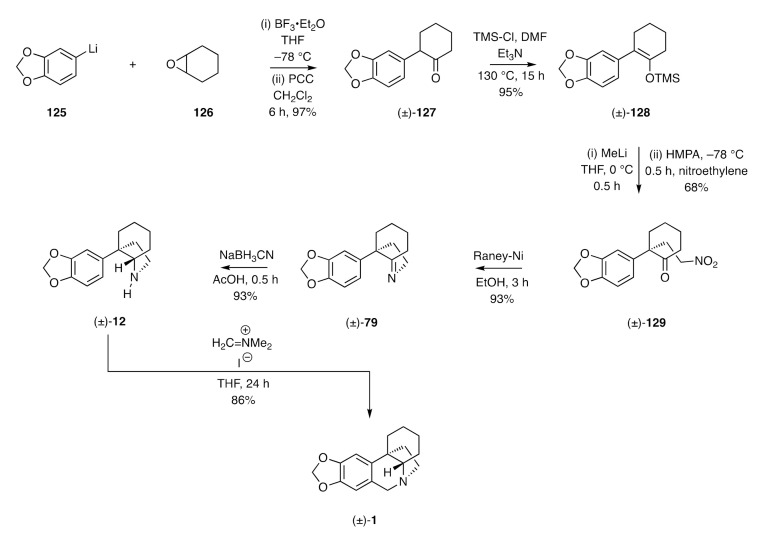
Tu’s synthesis of (±)-crinane [(±)-**1**].

α-Arylated cyclohexanones have proven to be useful precursors to the corresponding perhydroindoles required for the total synthesis of a range of crinine-type alkaloids and/or the parent framework (±)-**1**. An early illustration of the value of such precursors was provided by Tu [46], as shown in Scheme 17. Reaction of aryl lithium **125** with cyclohexene oxide (**126**) in the presence of BF_3_•Et_2_O afforded the anticipated product alcohol that could be oxidized to the ketone **127** using pyridinium chlorochromate (PCC). Conversion of this last compound into the corresponding silyl enol ether **128** under relatively standard conditions followed by treatment with methyl lithium and then nitroethylene afforded the Michael-type adduct **129**. Exposure of this compound to Raney–Nickel gave, via a reductive cyclization pathway, the cyclic imine (±)-**79**, a key intermediate associated with the Pearson synthesis of (±)-crinane (see Scheme 11). Reduction of the last compound afforded the corresponding 2°-amine (±)-**12**, which was treated with Eschenmoser’s salt to yield the target compound (±)-**1**.

**Scheme 18:**
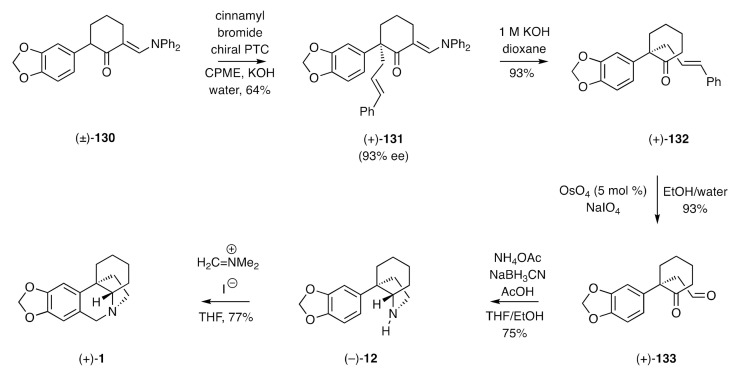
Maruoka’s asymmetric synthesis of (+)-crinane [(+)-**1**].

In a variation on the above theme, Maruoka and co-workers have described [47] the catalytic asymmetric alkylation of 2-arylcyclohexenones under phase-transfer conditions, thus enabling a synthesis of (+)-crinane [(+)-**1**]. The reaction sequence started (Scheme 18) with the key alkylation step that employed 1 mole % of a (*S*)-BINAP-based phase-transfer catalyst (PTC), the α-arylated, α’-*N*,*N*-diphenylaminomethylene-substituted cyclohexanone **130** as the substrate, cinnamyl bromide as the alkylating agent, and cyclopentyl methyl ether (CPME) as the solvent. By such means, the *S*-configured product **131** was obtained in 93% ee. Cleavage of the aminomethylene “auxiliary/blocking group” within this product was readily achieved using 1 M KOH, and the resulting α,α-disubstituted cyclohexanone **132** was subjected to oxidative cleavage of its cinnamyl side-chain. The resulting keto-aldehyde **133** engaged in a conventional reductive amination reaction to produce the C3a-arylated perhydroindole (−)-**12**, which was itself reacted with Eschenmoser’s reagent to give (+)-crinane [(+)-**1**].

**Scheme 19:**
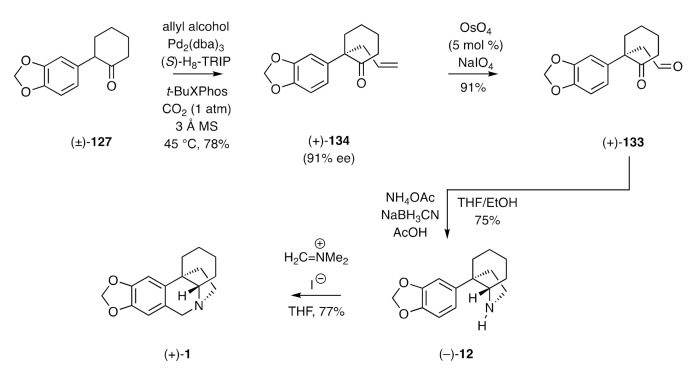
List’s asymmetric synthesis of (+)-crinane [(+)-**1**].

A closely related but slightly more direct route to (+)-crinane [(+)-**1**] was reported by List [48] and wherein (Scheme 19) the α-arylketone (±)-**127** was treated with allyl alcohol in the presence of Pd_2_(dba)_3_, *t*-BuXPhos, the chiral BINOL-derived phosphoric acid ligand (*S*)-H_8_-TRIP, and carbon dioxide to give, via a triple catalytic cycle involving Tsuji–Trost-type chemistry, the allylated compound (+)-**134** in 91% ee. Oxidative cleavage of this allylation product afforded keto-aldehyde (+)-**133**, which was carried forward, over two steps, in the same manner as used by Maruoka, to afford (+)-crinane [(+)-**1**].

**Figure 5:**
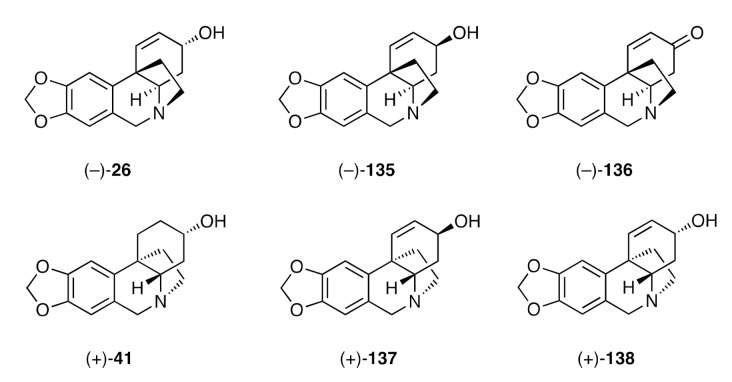
The structures of compounds (−)-**26**, (−)-**135**, (−)-**136**, (+)-**41**, (+)-**137**, and (+)-**138** prepared by Basai using a Pd-catalyzed enantioselective decarboxylative allylation of allylenol carbonates.

Similarly, Bisai and co-workers have described catalytic deacylative alkylations of enolcarbonates including for the generation of compound (±)-**134** (see above), thus allowing access to (±)-crinane [(±)-**1**] [49]. A catalytic asymmetric variant of such a process has been reported [33] by the same group and exploited in syntheses of (−)-crinine [(−)-**26**], (−)-*epi*-crinine [(−)-**135**], (−)-oxocrinine [(−)-**136**], (+)-*epi*-elwesine [(+)-**41**], (+)-vittatine [(+)-**137**], and (+)-*epi*-vittatine [(+)-**138**] (Figure 5).

**Scheme 20:**
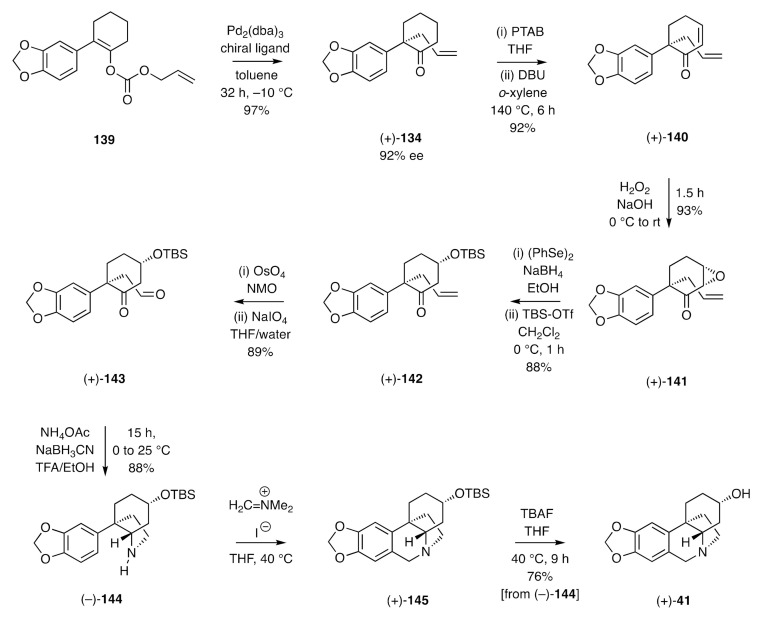
Bisai’s asymmetric synthesis of (+)-3-*epi*-elwesine [(+)-**41**].

The strategy involved is exemplified by the synthesis of (+)-*epi*-elwesine [(+)-**41**] as shown in Scheme 20 [50]. Thus, treatment of the allyl enol carbonate **139** with a palladium catalyst in the presence of a suitable chiral ligand resulted in a decarboxylative allylation reaction that afforded compound (+)-**134** in 92% ee. Treatment of the last compound with phenyl trimethylammonium tribromide (PTAB) and dehydrobromination of the resulting α-bromoketone using 1,8-diazabicyclo[5.4.0]undec-7-ene (DBU) as base gave enone (+)-**140**, which was subjected to nucleophilic epoxidation under standard conditions. The product epoxy ketone (+)-**141** was then subjected to reaction with in situ generated NaSePh and the resulting β-hydroxycyclohexanone silylated to give the *t*-butyldimethylsilyl (TBS) ether (+)-**142**. Oxidative cleavage of the terminal double bond within compound (+)-**142** afforded the keto-aldehyde (+)-**143**, and this was subjected to reductive amination conditions to afford the C3a-arylated perhydroindole (+)-**144**. This was, in turn, engaged in a Pictet–Spengler reaction using Eschenmoser’s salt, thus providing the TBS-ether (+)-**145** of the target crinine derivative (+)-**41**, which was itself formed through a desilylation reaction using tetra-*n*-butylammonium fluoride (TBAF).

The Basai group has also reported [51,52] a synthesis of (±)-crinane that involves 1,2-reduction of enone **53** (Scheme 8) to the corresponding allylic alcohol, engagement of this intermediate in an Eschenmoser–Claisen rearrangement, and conversion, using conventional manipulations, of the resulting unsaturated amide into keto-aldehyde (±)-**133** (Scheme 18). This last compound was finally converted into target (±)-**1** using the same protocols employed by Maruoka and List (Scheme 18 and Scheme 19, respectively).

**Scheme 21:**
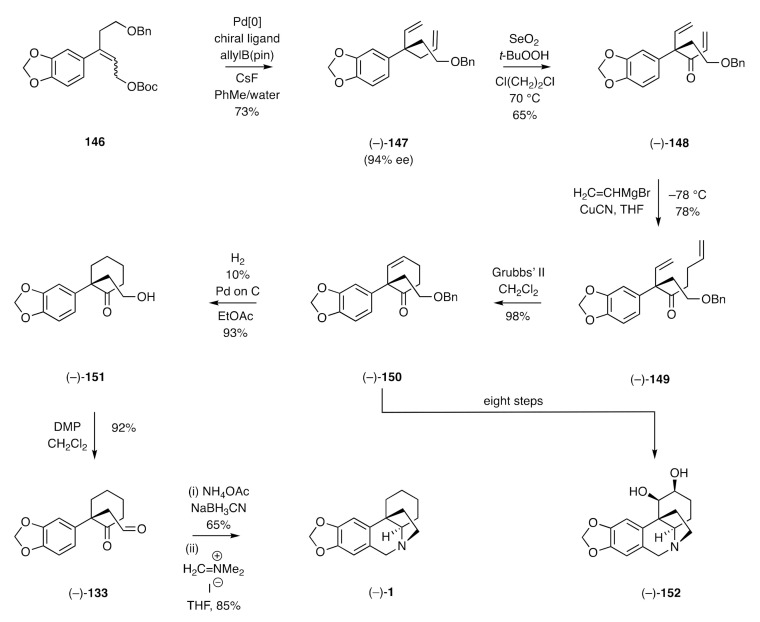
Wang’s syntheses of (−)-crinane [(−)-**1**] and (–)-4a-dehydroxycrinamabine [(–)-**152**].

The keto-aldehyde (−)-**133** has also featured in the synthesis of (−)-crinane reported by Wang [53], who has used the same methodology to establish a total synthesis of the naturally-occurring alkaloid (–)-4a-dehydroxycrinamabine. Details of the latter work are presented in Scheme 21. The pivotal and opening step involved an asymmetric Tsuji–Trost reaction employing the readily accessible styrene **146** (mixture of isomers) as the substrate and allylB(pin) as the nucleophile. When the reaction was carried out with a suitable chiral ligand and in the presence of caesium fluoride, then diene (−)-**147** was obtained in 94% ee. Allylic oxidation of compound (−)-**147** then provided enone (−)-**148** and conjugate addition of vinylmagnesium bromide to the latter afforded diene (−)-**149**. Subjection of this last compound to a ring-closing metathesis (RCM) using the Grubbs’ II catalyst afforded cyclohexenone (−)-**150**, which upon exposure to hydrogen in the presence of Pd on C was converted into the saturated alcohol (−)-**151**. Oxidation of the latter compound using the Dess–Martin periodinane (DMP) provided the by now familiar keto-aldehyde (−)-**133** that was converted, by the usual means, into (−)-crinane [(−)-**1**].

The C=C bond of intermediate (−)-**150** associated with the above sequence could be dihydroxylated stereoselectively under standard conditions and the diol so formed carried forward over a further seven steps, including a concluding Pictet–Spenger reaction, to deliver (–)-4a-dehydroxycrinamabine [(–)-**152**].

**Scheme 22:**
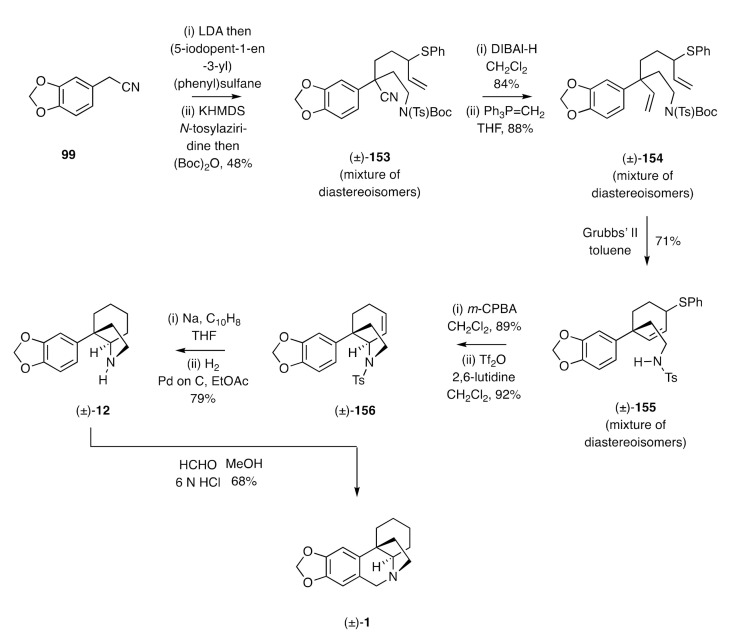
Raghavan’s synthesis of (−)-crinane [(−)-**1**].

Ring-closing metathesis served as a pivotal step in the Raghavan synthesis of (±)-crinane [(±)-**1**] [54], which started, as shown in Scheme 22, with the two-fold, base-promoted alkylation of nitrile **99**, using (5-iodopent-1-en-3-yl)(phenyl)sulfane and *N*-tosylaziridine as the electrophiles. Product (±)-**153** was thereby obtained as a mixture of diastereoisomers, and the associated nitrile residue was reduced with DIBAl-H to give, after hydrolysis, the corresponding aldehyde that was itself subject to a Wittig olefination reaction, thus forming the diene (±)-**154**. Subjection of this last compound to an RCM reaction using the Grubbs’ II catalyst then gave the cyclohexene (±)-**155**, which was engaged in an intramolecular S_N_′ reaction, with the sulfide residue being activated toward displacement by the sulfonamide nitrogen through initial *m*-chloroperbenzoic acid (*m*-CPBA)-mediated oxidation at sulfur. The product sulfoxides were then treated with triflic anhydride (Tf_2_O) in 2,6-lutidine to give hexahydroindole (±)-**156**. The tosyl group associated with this last compound was cleaved using sodium naphthalenide and the cyclohexene C=C bond then reduced under conventional conditions to deliver the C3a-aryl perhydroindole (±)-**12**. Pictet–Spengler cyclization of compound (±)-**12** was achieved using formaldehyde in the presence of HCl, thus affording (±)-crinane [(±)-**1**] in 68% yield.

**Scheme 23:**
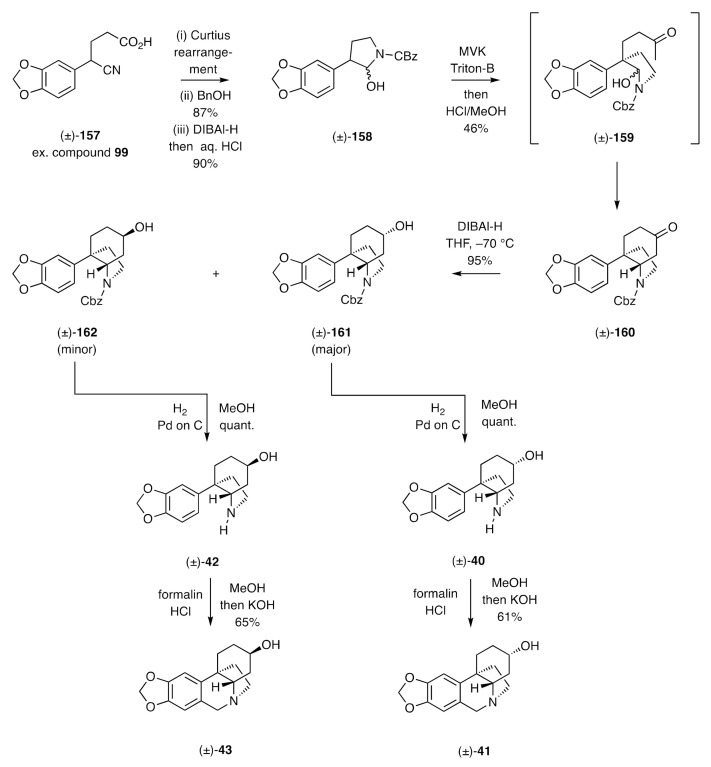
Sánchez’s syntheses of elwesine [(±)-**43**] and (±)-3-*epi*-elwesine [(±)-**41**].

Alkylation of nitrile **99** also served the key opening step in Sánchez’s syntheses [55] of elwesine [(±)-**43**] and 3-*epi*-elwesine [(±)-**41**]. The reaction sequence is shown in Scheme 23. The starting material, viz. compound **99**, was converted over four steps into the acid (±)-**157** and this itself subjected to a Curtius rearrangement. The resulting isocyanate was then trapped with benzyl alcohol, and the nitrile group appended to the ensuing carbamate was reduced with DIBAl-H to yield an imine that underwent hydrolysis and cyclization to deliver the pyrrolidine (±)-**158**. This was, in turn, treated with methyl vinyl ketone in the presence of the base Triton-B to afford, presumably via intermediate (±)-**159**, the Mannich product (±)-**160** incorporating the CD-ring substructure of the final targets. Reduction of the ketone carbonyl within this last compound using DIBAl-H then gave the epimeric mixture of alcohols (±)-**161** and (±)-**162** that could be separated chromatographically. Cleavage of the Cbz group associated with each of these epimers under standard hydrogenolytic conditions furnished the corresponding amino-alcohols (±)-**42** and (±)-**40**, respectively. The acquisition of these compounds constituted formal total syntheses of elwesine [(±)-**43**] and *epi*-elwesine [(±)-**41**] by virtue of their conversions, by Stevens (Scheme 6), into these targets under Pictet–Spengler conditions.

**Scheme 24:**
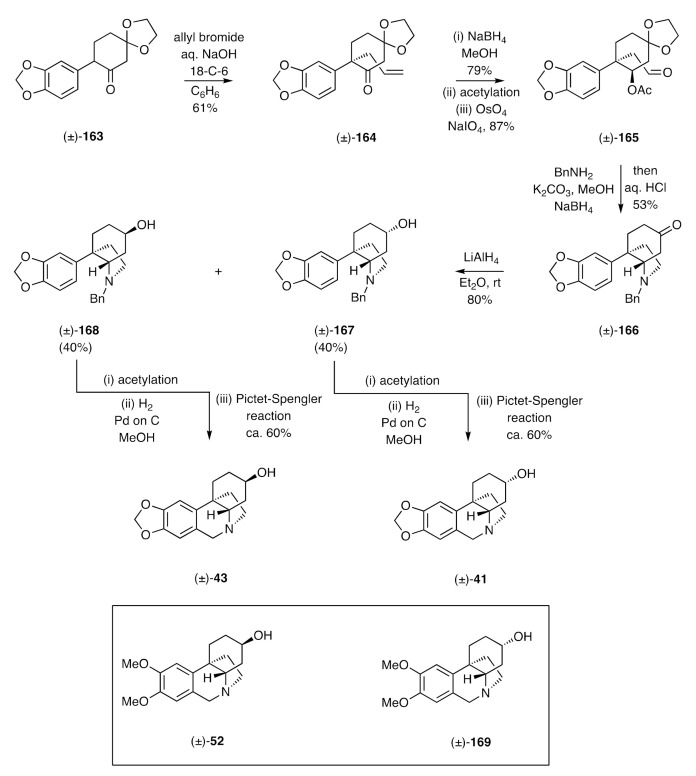
Umezawa’s syntheses of elwesine [(±)-**43**] and 3-*epi*-elwesine [(±)-**41**] and the structures of (±)-dihydromaritidine [(±)-**52**] and (±)-3-*epi*-dihydromaritidine [(±)-**169**], obtained by an analogous reaction sequence.

Umezawa and co-workers have also described [56] syntheses of elwesine [(±)-**43**] and 3-*epi*-elwesine [(±)-**41**]. Their route (Scheme 24) involved, as a key step, the alkylation of the α-arylated cyclohexane-1,4-dione monoethylene acetal (±)-**163** with allyl bromide using 50% aq. NaOH in the presence of 18-C-6. The product ketone (±)-**164** was stereoselectively reduced, using NaBH_4_, to the corresponding unsaturated alcohol that was itself acetylated under standard conditions. The double bond in the resulting ester was then subjected to oxidative cleavage, thus delivering the ester-aldehyde (±)-**165**. Reductive amination of this last compound using benzylamine in the presence of NaBH_4_ then gave, after treatment of the crude reaction product with aqueous HCl, the ketone (±)-**166**. Reduction, with LiAlH_4_, then delivered the corresponding and chromatographically separable epimeric alcohols, (±)-**167** and (±)-**168**, the acetate derivatives of which were subjected to hydrogenolytic cleavage using hydrogen in the presence of PdCl_2_ with methanol as solvent. Subjection of the products of this process to separate Pictet–Spengler reactions then gave elwesine [(±)-**43**] and 3-*epi*-elwesine [(±)-**41**]. A similar sequence of reactions starting with the dimethoxy-analogue of compound (±)-**163** afforded (±)-dihydromaritidine [(±)-**52**] and (±)-3-*epi*-dihydromaritidine [(±)-**169**].

**Scheme 25:**
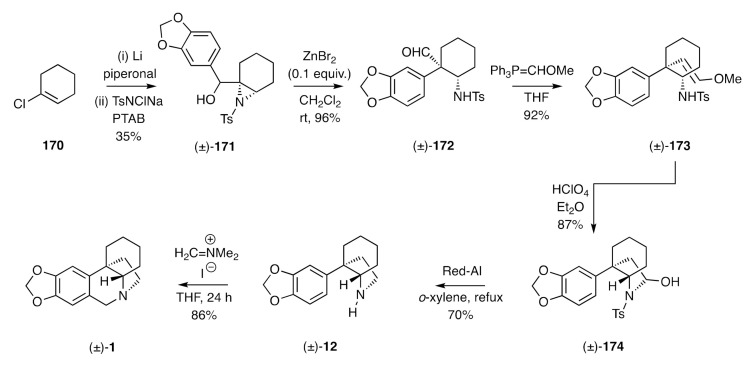
Tu’s synthesis of (±)-crinane via the Lewis acid-promoted rearrangement of a 2,3-aziridino alcohol.

A synthesis of (±)-crinane using a Lewis-acid promoted rearrangement of a 2,3-aziridino alcohol has been reported by Tu [57] (Scheme 25). Thus, successive treatment of chlorocyclohexene **170** with lithium and piperonal followed by reaction of the ensuing allylic alcohol with chloramine-T (TsNClNa) and phenyltrimethylammonium tribromide (PTAB) afforded the 2,3-aziridino alcohol (±)-**171**. Upon exposure of this intermediate to zinc bromide, a smooth rearrangement took place to afford the isomeric aldehyde (±)-**172**. Wittig reaction of this last compound afforded the enol ether (±)-**173** and upon treatment of this product with aqueous perchloric acid in diethyl ether, hydrolysis then cyclization took place to give the 2-pyrrolidinol (±)-**174**. Reaction of this last compound with NaAlH_2_(OCH_2_CH_2_OCH_3_)_2_ (Red-Al) at elevated temperatures resulted in reductive cleavage of both the hydroxyl and tosyl residues to afford the C3a-arylated perhydroindole (±)-**12**, a well-established precursor to (±)-crinane [(±)-**1**)].

**Scheme 26:**
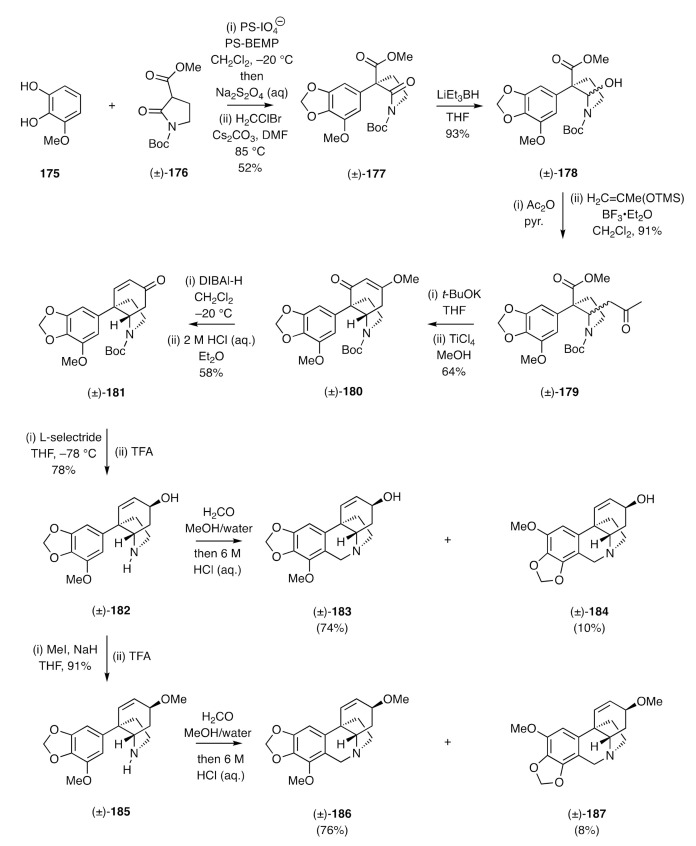
The Dixon syntheses of (±)-powelline [(±)-**183**] and (±)-buphanidrine [(±)-**186**].

Arylated 2-pyrrolidinols feature in the syntheses of (±)-powelline and (±)-buphanidrine reported by Dixon and co-workers [58,59]. The pivotal and opening feature of the reaction sequence (Scheme 26) was the oxidative coupling of catechol **175** with the acylated and *N*-Boc protected lactam (±)-**176** using polymer supported (PS) periodate in the presence of the similarly supported base 2-*t*-butylimino-2-diethylami -no-1,3-dimethylperhydro-1,3,2-diaza-phosphorine (BEMP). By this means and after alkylation of the product catechol with bromochloromethane (to install the methylenedioxy unit), the arylated lactam (±)-**177** was obtained. Reduction of the lactam carbonyl moiety within compound (±)-**177** then gave the arylated 2-pyrrolidinol (±)-**178**, which could be alkylated with the TMS-enol ether of acetone in the presence of a Lewis acid to afford the methyl ketone (±)-**179**. This keto-ester was engaged in a Dieckmann-type cyclization using potassium *t*-butoxide, and the product cyclohexane-1,3-dione reacted with methanol in the presence of TiCl_4_ to give the β-methoxy-substituted cyclohexenone (±)-**180** that was itself reduced with DIBAl-H and, after acidic work-up, yielded the new enone (±)-**181**. Stereoselective 1,2-reduction of this last compound was achieved using L-selectride, and the Boc group associated with the product allylic alcohol (±)-**181** was cleaved using TFA to give the 2°-amine (±)-**182**. Subjection of this last compound to a Pictet–Spengler reaction using aqueous formaldehyde in methanol followed by treatment with 6 M aqueous HCl then gave a mixture of (±)-powelline [(±)-**183**] and its regio-isomeric cyclization product (±)-**184**. The synthesis of (±)-buphanidrine involved (Scheme 26) the *O*-methylation of allylic alcohol (±)-**182** under standard conditions. The resulting ether was treated with TFA to give compound (±)-**185**, which on being subjected to a Pictet–Spengler reaction again afforded the target alkaloid (±)-**186** [viz. (±)-buphanidrine] and its regioisomer (±)-**187**.

**Scheme 27:**
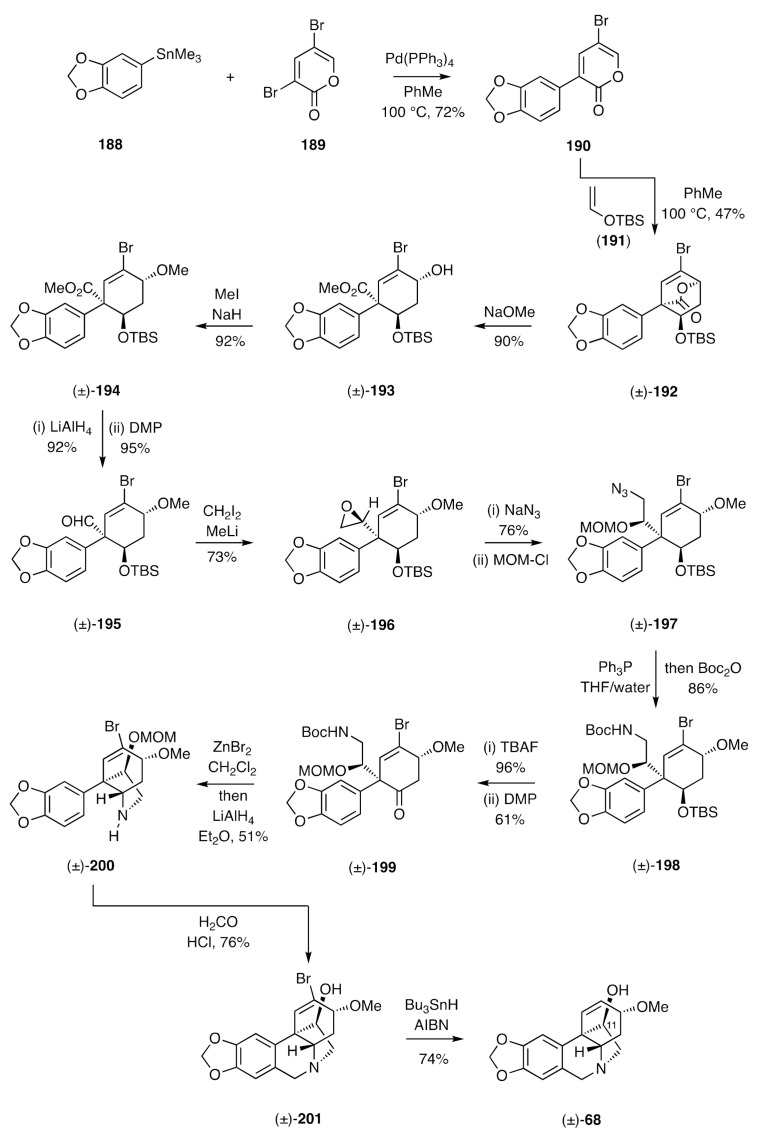
The Cho synthesis of (±)-crinamine [(±)-**68**].

Cho’s synthesis of (±)-crinamine [60] is distinctive because of the less “obvious” nature of the starting materials and the capacity it provides to introduce oxygenation at the enthano-bridge of the crinane framework, a structural feature encountered in a range of such natural products including (±)-crinamine [compound (±)-**68**, see Scheme 9]. The reaction sequence used is shown in Scheme 27 and starts with the Stille cross-coupling of stannane **188** with the dibromo-α-pyrone **189**, followed by engagement of the product **190** in an inverse electron-demand Diels–Alder reaction with the enol ether **191** to afford the *endo*-adduct (±)-**192** as the major product of reaction. Cleavage of the lactone bridge within the last compound was effected using sodium methoxide and the product hydroxyl-ester (±)-**193** was *O*-methylated to give ether (±)-**194**. Lithium aluminium hydride-mediated reduction of the associated ester group gave the corresponding alcohol, which was converted into aldehyde (±)-**195** on reaction with DMP. Reaction of this last compound with the anion derived from di-iodomethane and methyl lithium then gave epoxide (±)-**196**. This was opened with sodium azide and the product azido-alcohol protected as the corresponding MOM-ether (±)-**197**. A Staudinger reaction followed by protection of the product amine as its Boc-derivative then gave compound (±)-**198** that was itself treated with TBAF and the resulting alcohol oxidized to ketone (±)-**199** using DMP. A transannular reductive amination, to install what would become the ethano-bridge in the final product, was then achieved by sequential treatment of the last compound with zinc bromide and then lithium aluminium hydride to produce the C3a-arylated perhydroindole (±)-**200**. A Pictet–Spengler reaction followed, thus producing the bromo-derivative, (±)-**201**, of the final target. A radical-based reductive debromination reaction using tri-*n*-butyltin hydride in the presence of AIBN then gave (±)-crinamine [(±)-**68**].

Using closely related chemistry, Cho and his co-workers could exploit compound (±)-**193** in syntheses of (±)-crinine [(±)-**26**] and (±)-11-*epi*-crinamine (structure not shown).

**Scheme 28:**
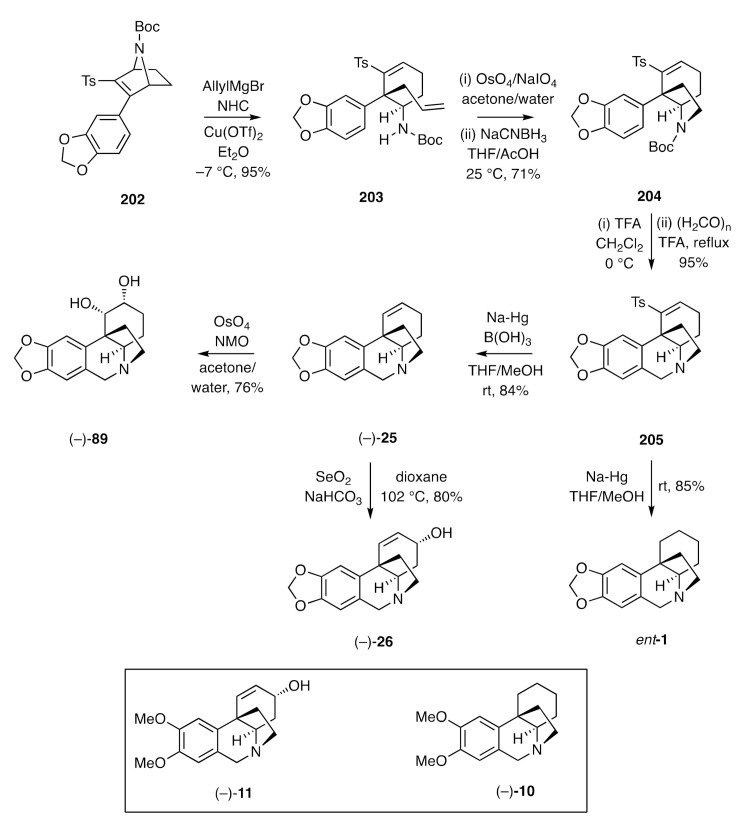
Pandey’s syntheses of (−)-amabiline [(−)-**89**], (−)-crinine [(−)-**26**], and (−)-crinane [*ent*-**1**] and the structures of (−)-maritidine [(−)-**11**] and (−)-oxomaritidine [(−)-**10**] obtained by an analogous reaction sequence.

Pandey and co-workers have also employed Diels–Alder-derived and bridged bicyclic compounds as starting materials in the synthesis of certain crinine alkaloids [61]. The starting point for their work was the homochiral 7-azabicyclo[2.2.1]heptane **202** (Scheme 28) obtained through the reaction of *N*-Boc protected pyrrole with a suitably substituted acetylene. Reaction of compound **202** with allylmagnesium bromide in the presence of a *N*-heterocyclic carbene (NHC) catalyst and copper triflate gave the ring-opened product **203**. The terminal C=C bond within this compound was oxidatively cleaved, and the aldehyde so-formed underwent Schiff-base condensation with the pendant carbamate nitrogen. The product imine was then reduced to afford the C3a-arylated hexahydroindole **204**. Reaction of compound **204** with TFA and subjection of the 2°-amine so-formed to a Pictet–Spengler reaction then gave 3°-amine **205**, which on treatment with sodium amalgam in the presence of boric acid afforded the olefinic reduction product (−)-**25**. Dihydroxylation of the latter intermediate (under standard conditions) then gave (−)-amabiline [(−)-**89**]. Alternatively, allylic oxidation of compound (−)-**25** afforded (−)-crinine [(−)-**26**], while exhaustive reduction of precursor **205** gave the parent ring system *ent*-**1** in homochiral form. By using an analogous set of transformations starting with the dimethoxy-analogue of compound **202** then (−)-maritidine [(−)-**11**] and (−)-oxomaritidine [(−)-**10**] could also be obtained.

**Scheme 29:**
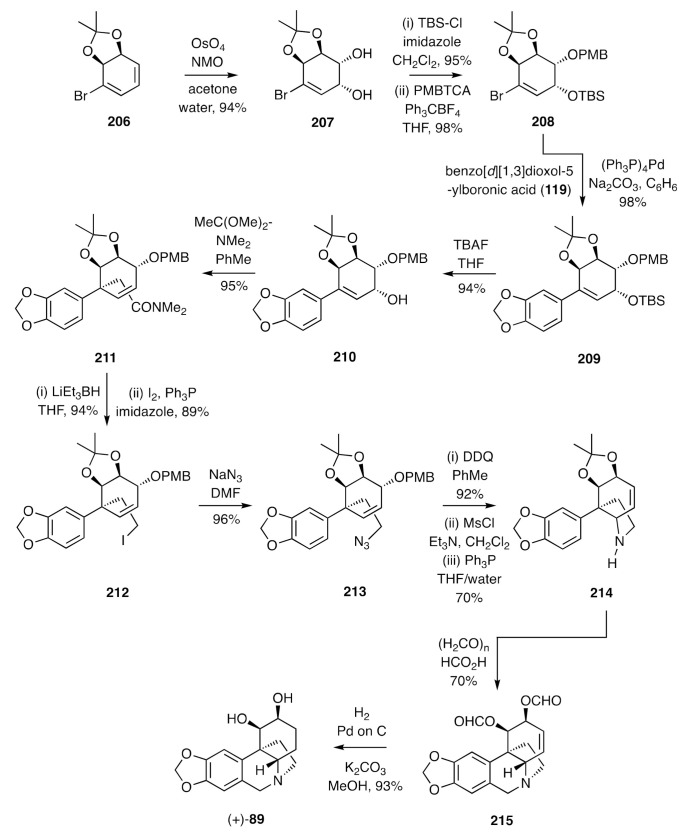
Findlay’s chemoenzymatic synthesis of (+)-amabiline [(+)-**89**].

Findlay established a chemoenzymatic total synthesis of (+)-amabiline [(+)-**89**] by the pathway shown in Scheme 29 [62]. This started with the homochiral acetonide **206** itself being generated from the corresponding diol (*cis*-1,2-dihydrocatechol) that is available in kilogram quantities through a whole-cell biotransformation of bromobenzene involving the toluene dioxygenase (TDO) enzyme [63]. Regio- and stereo-selective dihydroxylation of compound **206** gave the conduritol **207**, the hydroxyl groups of which could be selectively protected to form compound **208**. This last compound served as a substrate for a Suzuki–Miyaura cross coupling reaction with the commercially available benzo[*d*][1,3]dioxol-5-ylboronic acid (**119**), thus providing compound **209**. Reaction of the TBS ether-containing compound **209** with TBAF afforded the corresponding allylic alcohol **210**, which readily participated in an Eschenmoser–Claisen rearrangement reaction to afford amide **211**. Upon reduction of this amide and treatment of the product alcohol with iodine in the presence of triphenylphosphine, then halide **212** was obtained, and this was converted into the corresponding azide **213** under standard conditions. Reaction of this last compound with 2,3-dichloro-5,6-dicyano -1,4-benzoquinone (DDQ) resulted in cleavage of the *p*-methoxybenzyl (PMB) group, and the ensuing alcohol was then converted into the corresponding mesylate under standard conditions. The azide group associated with this ester was subjected to a Staudinger reaction using triphenylphosphine in aqueous THF and the 1°-amine so-formed engaged in spontaneous S_N_′ reaction to generate compound **214** that served as a substrate for a Pictet–Spengler reaction. Under the conditions used, the acetonide moiety associated with this substrate was also cleaved, and the hydroxyl groups so revealed were formylated to generate the diester **215**. Exposure of a methanolic solution of this last compound to hydrogen in the presence of both palladium on carbon and potassium carbonate resulted in concurrent reduction of the cyclohexene double-bond and cleavage of the ester moieties and thereby delivering (+)-amabiline [(+)-**89**].

**Scheme 30:**
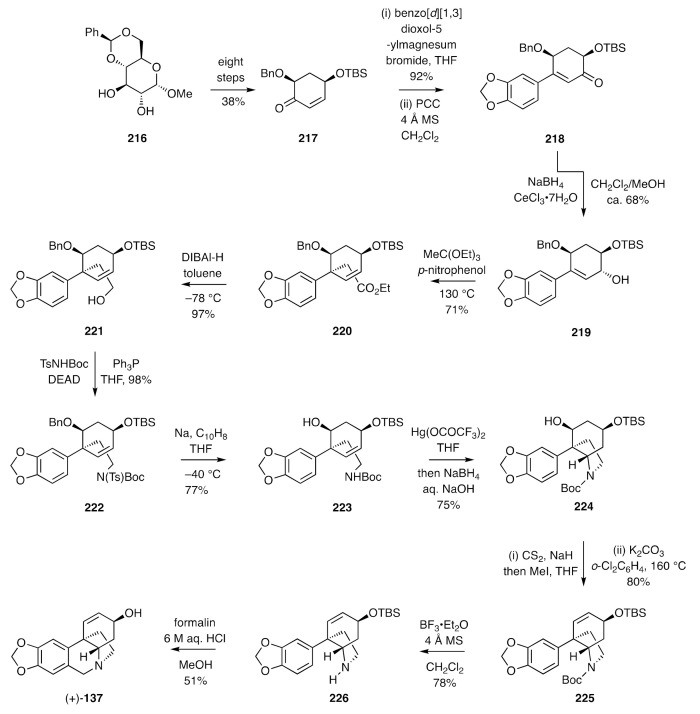
Chida’s synthesis of (+)-vittatine [(+)-**137**] from the glucose derivative **216**.

**Figure 6:**
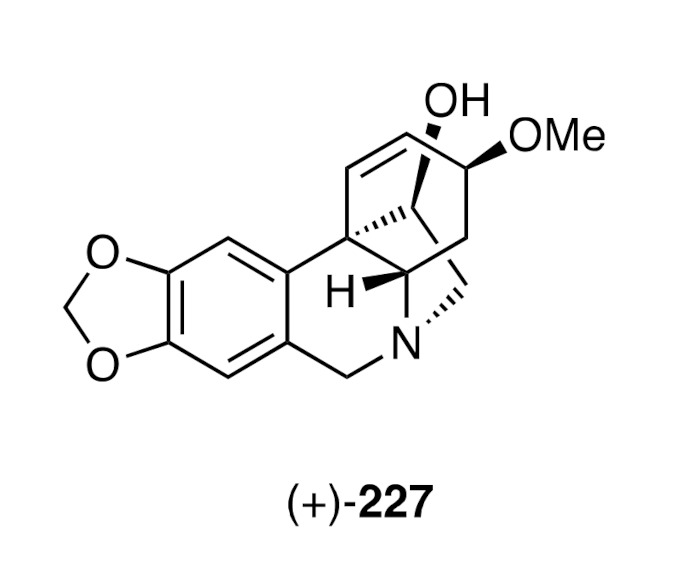
The structure of (+)-haemanthamine [(+)-**227**], an alkaloid prepared by Chida from the *D*-glucose derivative **216**.

**Scheme 31:**
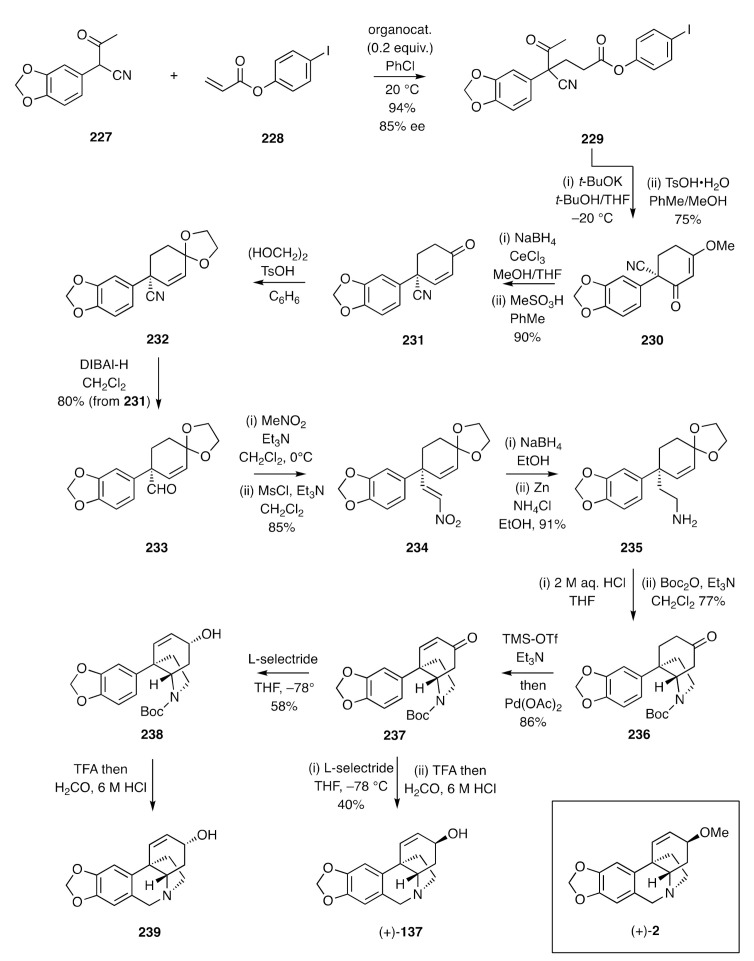
Fan’s synthesis of (+)-vittatine [(+)-**137**] and its epimer **239** together with the structure of (+)-buphanisine [(+)-**2**] prepared by related means.

In a related chiron-based approach, Chida has reported [64] syntheses of (+)-vittatine and (+)-haemanthamine from *D*-glucose. The first phase of this approach involved the conversion of methyl 4,6-*O*-benzylidene-α-d-glucopyranoside (**216**) (Scheme 30), a material available from multiple commercial suppliers, over eight steps including a Ferrier carbocyclization, into the homo-chiral enone **217**. Addition of the Grignard reagent derived from 5-bromobenzo[*d*][1,3]dioxole to the carbonyl group of compound **217** and treatment of the resulting 3°-alcohol with pyridinium chlorochromate (PCC), resulting in a Dauben–Michno oxidative rearrangement, gave the β-arylated enone **218**. This intermediate was subjected to stereoselective Luche reduction, thus affording allylic alcohol **219**, which could then be engaged in a Johnson–Claisen rearrangement, thus delivering ester **220**. Reduction of the last compound using DIBAl-H gave the alcohol **221**, which was engaged in a Mitsunobu reaction using *t*-butyl tosylcarbamate as the nucleophile. The product **222** so-formed was then reduced with sodium naphthalenide to give the Boc-protected amine **223**. An intramolecular amino-mercuration/de-mercuration protocol was then employed to assemble the C3a-arylatedperhydroindole **224**. The xanthate ester derived from the alcohol associated with the last compound was subjected to a pyrolytic *syn*-elimination reaction, and the carbamate residue embodied within the product olefin **225** cleaved with BF_3_•Et_2_O to give the 2°-amine **226**. Subjection of compound **226** to Pictet–Spengler conditions, under which the TBS-ether was also cleaved, then gave (+)-vittatine [(+)-**137**]. Related chemistry, including an end-game similar to that employed in the Cho synthesis of (±)-crinamine [(±)-**68**] (Scheme 27), also allowed for the preparation of (+)-haemanthamine [(+)-**227**] (Figure 6) from *D*-glucose.

(+)-Vittatine [(+)-**137**] was also synthesized by Fan [65] using an enantioselective organocatalytic Michael addition reaction as the opening and key step of the reaction sequence (Scheme 31). Thus, Michael addition of compound **227** to acrylate **228** in the presence of a quinidine-derived organo-catalyst afforded adduct **229** in 85% ee, which could be raised to 99% through recrystallization. Subjection of this product to a base-promoted Dieckmann-type condensation and treatment of the resulting 1,3-diketone with TsOH•H_2_O in the presence of methanol afforded the enone **230**, Luche reduction of which followed by acid treatment gave compound **231**. The associated ketone group of the last compound was then protected as the corresponding ethylene ketal **232**. DIBAl-H-mediated reduction of the nitrile unit within compound **232** gave, after work-up, the aldehyde **233**, and reaction of this product with nitromethane in the presence of triethylamine gave a nitro-alcohol that was dehydrated through treatment with methanesulfonyl chloride in the presence of triethylamine. The nitro-olefin **234** so formed was reduced to the corresponding 1°-amine **235**, and upon treatment of this with aqueous acid, ketal hydrolysis took place, and the resulting enone reacted with the pendant amine unit to give the expected C3a-arylated perhydroindole that was itself treated with Boc_2_O to give carbamate **236**. Dehydrogenation of the cyclohexanone unit within compound **236** was achieved under Saegusa–Ito conditions, and the enone so formed reduced in a 1,2-manner with L-selectride to give allylic alcohol **238** as the major product (58%), which was readily separated from its chromatographically less polar epimer (obtained in 40% yield). Cleavage of the Boc-group within compound **238** was achieved using TFA, and subjection of the ensuing 2°-amine to a Pictet–Spengler reaction then gave (+)-3-*epi*-vittatine [(+)-**239**], while an analogous two-step process applied to the epimer afforded (+)-vittatine [(+)-**137**] itself. Additionally, when the mesylates derived from the mixture of compound **238** and its epimer were subjected to methanolysis and the resulting allylic ether treated with TFA followed by formaldehyde and aqueous HCl, then (+)-buphanisine [(+)-**2**] was obtained stereoselectively and in good overall yield.

**Scheme 32:**
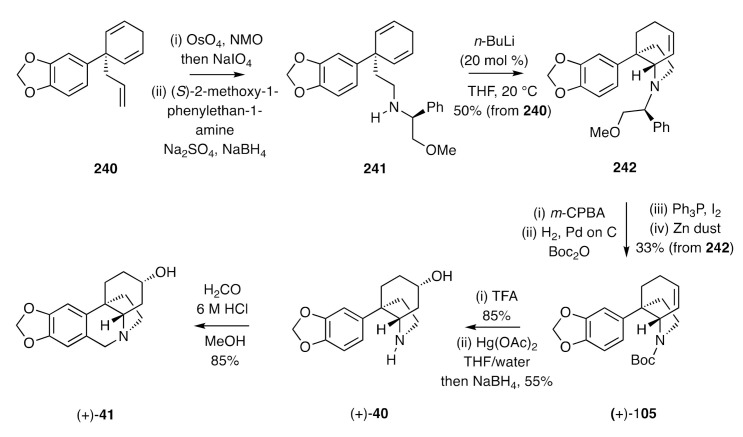
The Landais synthesis of (+)-3-*epi*-elwesine [(+)-**41**].

The based-catalysed intramolecular hydroamination of 1-ethylaminocyclohexa -2,5-dienes developed by Landais [66] has provided a useful method for the assembly of the crinine alkaloid framework as exemplified by the synthesis of (+)-3-*epi*-elwesine [(+)-**41**] (Scheme 32). Thus, the arylated cyclohexa-1,4-diene **240**, which can be prepared in a high-yielding five-step sequence from cyclohexane-1,3-dione, was subjected to selective oxidative cleavage and the product aldehyde reductively aminated using (*S*)-2-methoxy-1-phenyethan-1-amine, thus affording compound **241**, the substrate required for the pivotal intramolecular hydroamination reaction. On treating this 2°-amine with 20 mol % *n*-BuLi, conversion into the C3a-arylated hexahydroindole **242** was achieved. In order to remove the chiral auxiliary from this compound without concomitant reduction of the associated cyclohexene C=C bond, the olefin was protected as the corresponding epoxide, the auxiliary was then removed hydrogenolytically, and the resulting free amine protected as the corresponding *O*-*t*-butyl carbamate. Re-establishing the C=C bond required ring cleavage of the epoxide using Ph_3_P and molecular iodine and treatment of the resulting iodohydrin with zinc dust, thereby affording compound (+)-**105**. Following the protocols established in the closing stages of Overman’s synthesis of (±)-3-*epi*-elwesine (Scheme 14), the Boc-group within the last compound was removed using TFA, and the cyclohexene double bond was subjected to an oxymercuration/demercuration sequence that stereoeselectively produced alcohol (+)-**40**. Upon subjection of this material to a traditional Pictet–Spengler reaction, then (+)-3-*epi*-elwesine [(+)-**41**] was obtained.

**Scheme 33:**
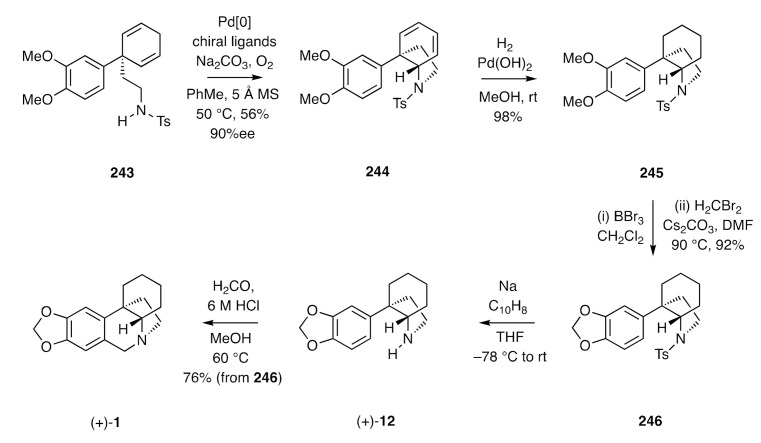
Zhu’s synthesis of (+)-crinane [(+)-**1**].

More recently, Zhu has described [67] a desymmetrizing aza-Wacker-based variant of the Landais protocol that allowed for the synthesis of (+)-crinane [(+)-**1**]. The starting material used in this synthesis (Scheme 33) was the diene **243** obtained through selective Birch reductive alkylation of the corresponding biaryl. Exposure of compound **243** to a palladium catalyst and certain chiral ligands in the presence of sodium carbonate and oxygen gave the C3a-arylated tetrahydroindole **244** in 90% ee. Reduction of the cyclohexadiene C=C bonds was achieved using hydrogen in the presence of Pearlman’s catalyst, and the two methoxy groups associated with product **245** were manipulated by standard methods to install the corresponding methylenedioxy unit as seen in congener **246**. Cleavage of the tosyl group within the last compound then gave the perhydroindole (±)-**12**, which was converted into (+)-crinane [(±)-**1**] under standard Pictet–Spengler conditions.

**Scheme 34:**
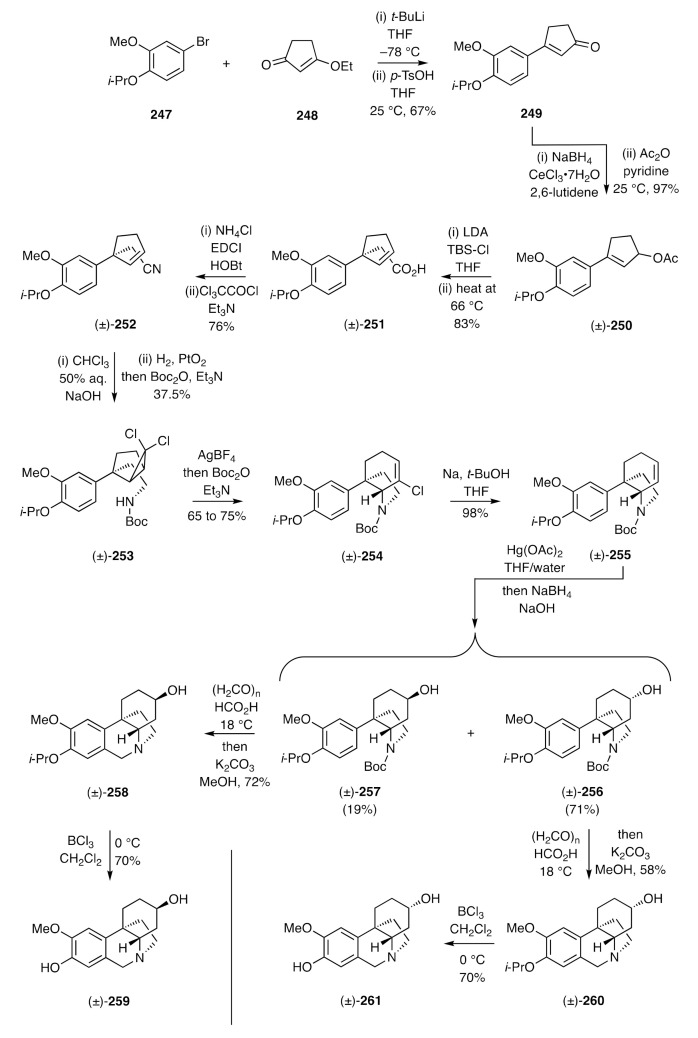
Harvey’s synthesis of (±)-maritinamine [(±)-**259**] and (±)-3-*epi*-maritinamine [(±)-**261**].

As highlighted in Scheme 16, while cyclopropane ring-expansion reactions provide a means for assembling the crinane framework, the radical cyclization reaction required to install the D-ring in that approach does not allow for functionalization of the C-ring. A related approach that circumvents such deficiencies is highlighted in Harvey’s synthesis of maritinamine and its C3-epimer [68]. The reaction sequence started, as shown in Scheme 34, with the conversion of the aryl bromide **247** into the corresponding lithio-species that then added, in a 1,2-manner, to the enone **248**, thus affording, after acidic work-up, compound **249**. Luche reduction of the last species and acetylation of the ensuing allylic alcohol then provided the unsaturated acetate (±)-**250**, which readily engaged in an Ireland–Claisen rearrangement reaction to afford carboxylic acid (±)-**251**. This acid was readily converted into the nitrile (±)-**252** and this, in turn, subjected to reaction with dichlorocarbene generated under phase transfer conditions. As a result, a 2:1 mixture of diastereoisomeric carbene adducts was obtained, and each of these could be converted into the corresponding carbamate [e.g., (±)-**253**] under standard conditions. Each of these was, in turn, independently engaged in a silver ion induced electrocyclic ring-opening reaction with the ensuing π-allyl cation then being trapped nucleophilically by the pendant carbamate residue, thus delivering the same C3a-arylated hexahydroindole, namely (±)-**254**, in 65–75% yield. Reductive dechlorination of the last compound was achieved using sodium in *t*-butanol and the resulting alkene subjected to an oxy-mercuration/demercuration reaction sequence, thus delivering a ca. 3:1 mixture of the epimeric alcohols (±)-**256** and (±)-**257**. Each of these was subjected to a Pictet–Spengler reaction conducted under standard conditions with the products (±)-**258** and (±)-**260**, each being treated with BCl_3_ to effect cleavage of the associated *i*-propyl aryl ether residues, thus delivering (±)-maritinamine [(±)-**259**] and (±)-*epi*-maritinamine [(±)-**261**], respectively.

**Scheme 35:**
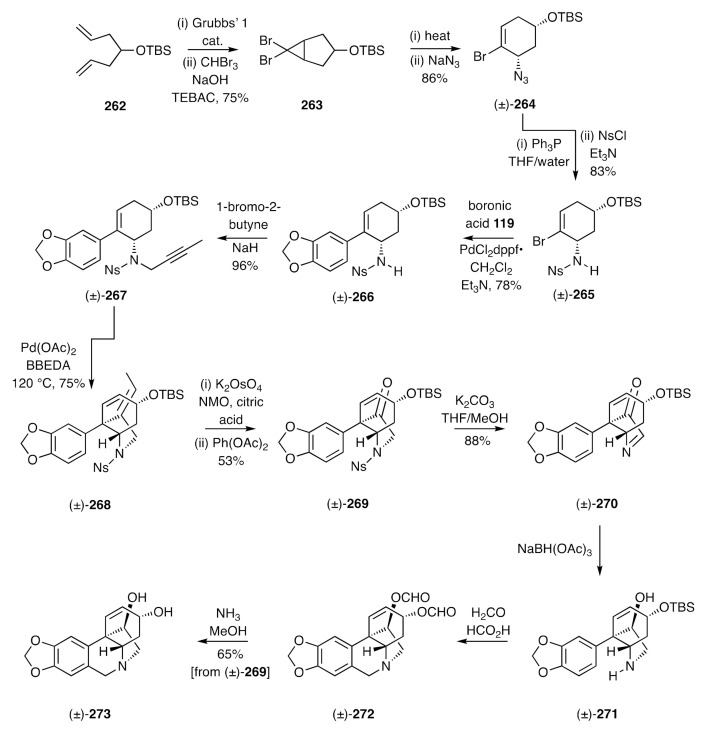
Petit’s synthesis of (±)-hamayne [(±)-**273**].

A related approach (Scheme 35) has been employed by Petit [69] in the synthesis of the alkaloid hamayne that incorporates a hydroxyl group at C11 of the ethano-bridge. The synthesis began with the RCM reaction of the readily available diene **262** and dibromocarbene addition to the product cyclopentene to afford a diastereoisomeric mixture of adducts **263**, the major one being readily obtained in pure form and 75% yield. Heating this major adduct resulted in electrocyclic ring-opening of the cyclopropane ring and formation of the isomeric dibromocyclohexene, the allylically-positioned halogen of which was displaced with sodium azide to give compound (±)-**264**. Staudinger reduction of this azide and reaction of the product amine with nosyl chloride (Ns-Cl) gave the sulfonamide (±)-**265**, which was engaged in a Suzuki–Miyaura cross coupling reaction with commercially available benzo[d][1,3]dioxol-5-ylboronic acid (**119**) to afford styrene (±)-**266**. *N*-Alkylation of this last compound with 1-bromo-2-butyne in the presence of sodium hydride then gave compound (±)-**267** that could be enaged in a palladium-catalyzed intramolecular Alder-ene (IMAE) reaction, using *bis*-benzylidene ethylenediamine (BBEDA) as ligand, to generate the C3a-arylated hexahydroindole (±)-**268**. Some levels of selectivity could be achieved in preferentially dihydroxylating the tri-substituted (and exocyclic) double bond within this diene, and the product diol was then oxidatively cleaved to give ketone (±)-**269**. Reaction of this last compound with potassium carbonate resulted in an E1_cb_ reaction, and the product imine (±)-**270** was reduced in a stereoselective manner to give the alcohol (±)-**271**, which was itself subjected to a Pictet–Spengler reaction. As a result, the di-formate (±)-**272** was obtained, and upon exposure of this compound to ammonia in methanol, the associated ester residues were cleaved and hamayne [(±)-**273**] thereby obtained.

**Figure 7:**
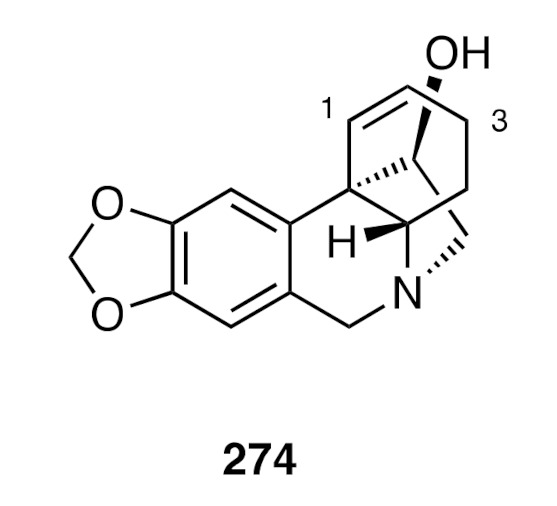
The structure assigned to the alkaloid haemultine (**274**), the racemate of which was prepared using a variation on the route shown in Scheme 35.

A closely related protocol was employed [70] to prepare compound (±)-**274** (Figure 7), the C3-deoxy analogue of hamayne and the structure assigned, in a 1958 report, to the alkaloid haemultine. This alkaloid was isolated from the colorful bush lily *Haemanthus multiflorus*, extracts of which are used in certain traditional African medicines. The racemate (±)-**274** could be resolved into its constituent enantiomers through formation of the corresponding Mosher esters, and after separation of the resulting diastereoisomers, these could be converted, under reducing conditions, into enantiomers (+)-**274** and (−)-**274**. The physical and spectroscopic data derived from the former enantiomer were consistent with the proposition that the naturally-derived material was, in fact, a mixture of the crinine (+)-**274** and its Δ^2,3^-double bond isomer.

**Scheme 36:**
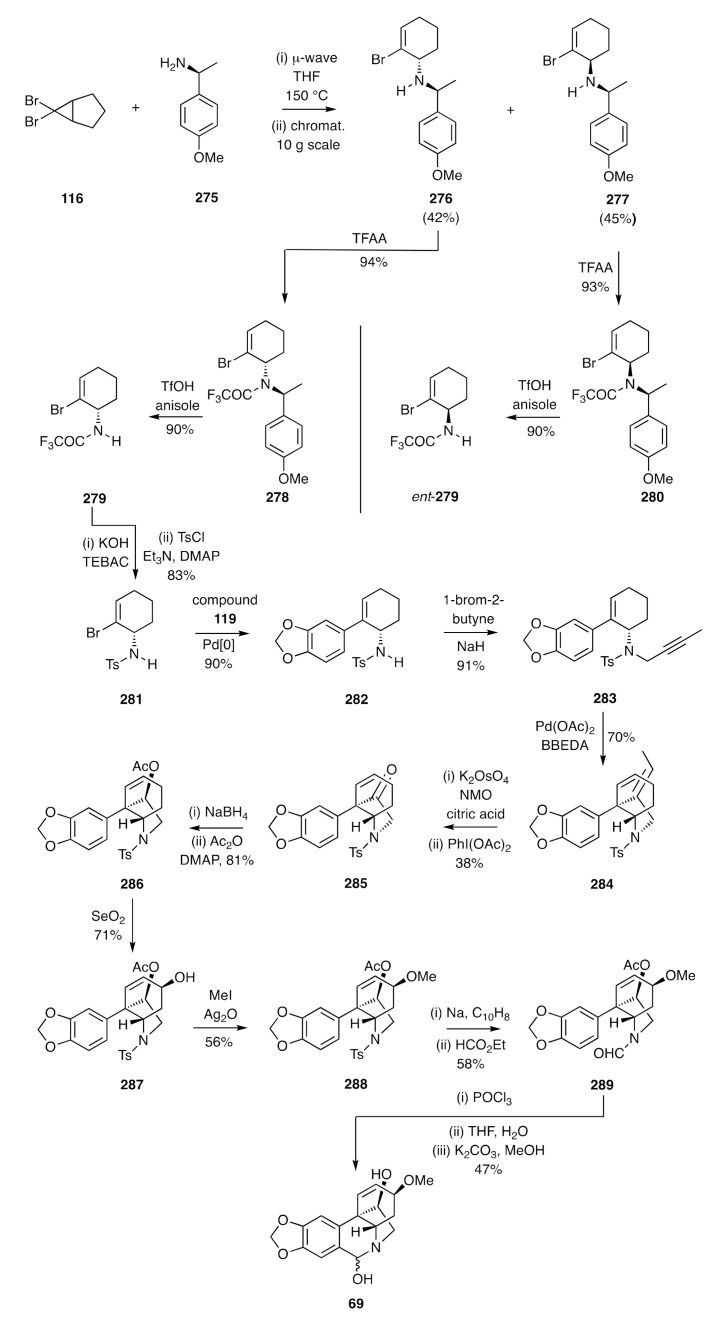
Lan’s synthesis of (−)-haemanthidine [(−)-**69**].

The strategy shown in Scheme 34 and Scheme 35 could also be adapted, as shown by Lan [71], to the synthesis of certain enantiomerically pure crinine alkaloids. If, for example, cyclopropane **116** was subjected to microwave-induced electrocyclic ring-opening and the ensuing π-allyl cation captured with the homochiral and commercially available 1°-amine **275**, then the anticipated pair of diastereoisomeric adducts **276** and **277** was obtained (Scheme 36). These could be readily separated by conventional chromatographic techniques at multi-gram scale, and each reacted with trifluoracetic anhydride (TFAA) to give the corresponding trifluoroacetamide **278** and **280**, respectively. The PMB groups associated with each of these could, in turn, be cleaved with triflic acid (TfOH) when using anisole as a scavenging agent to intercept the benzyl cations so-formed. The resulting and enantiomerically related amides **279** and *ent*-**279** were thus obtained. The former compound was subject to hydrolysis and the homochiral product amine then converted, under standard conditions, into the corresponding toluenesufonamide **281**. Suzuki–Miyaura cross-coupling of this cyclohexenyl bromide with boronic acid **119** then gave styrene **282** and this was *N*-propagylated, via reaction with 1-bromo-2-butyne in the presence of NaH, to afford sulfonamide **283**. The last compound proved to be a competent substrate for an IMAE reaction that was effected using Pd(OAc)_2_ and delivered the C3a-arylated hexahydroindole **284**. A two-step oxidative-cleavage of the exocyclic double-bond within this diene then gave ketone **285**, which upon reduction with sodium borohydride and acetylation of the product alcohol gave the unsaturated ester **286**. Subjection of this ester to allylic oxidation using SeO_2_ afforded alcohol **287**, which was converted into the corresponding methyl ether **288** under Irvine–Purdie conditions. Reductive cleavage of the sulfonamide residue within this last compound was accomplished using sodium naphthalenide and the product amine treated with ethyl formate, thus generating the formamide **289**. In the final stage of the synthesis, compound **289** was treated with POCl_3_ to effect a Bischler–Napieralski rather than a Pictet–Spengler cyclization and thereby provided, after work-up steps, the target (−)-haemanthidine [(−)-**69**] as an interconverting mixture of diastereoisomers.

The utility of this approach was emphasized through its deployment in total syntheses of (+)-haemanthidine, (+)- and (−)-crinane, (+)- and (−)-11-hydroxyvattitine, (+)- and (−)-11-bulbispermine, and (±)-hamayne and (±)-apohaemanthamine [71].

**Scheme 37:**
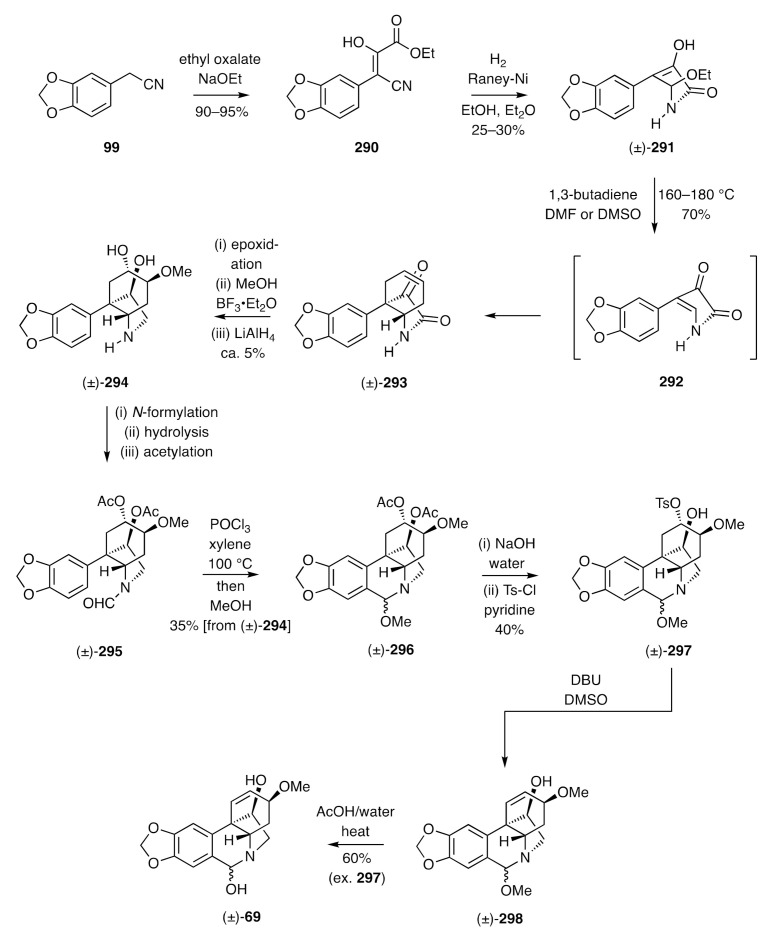
Tsuda’s synthesis of (±)-haemanthidine [(±)-**69**].

The end-game associated with the total synthesis described immediately above was inspired by Tsuda’s synthesis of racemic haemanthidine [(±)-**69**] as shown in Scheme 37 [72,73]. This started with the reaction of the nitrile **99** and ethyl oxalate in the presence of sodium ethoxide to give the cinnamate **290**. Reductive cyclization of this intermediate using hydrogen in the presence of Raney nickel afforded lactam (±)-**291**. On heating this last compound at elevated temperatures, ethanol was expelled and the product enamine **292** trapped in situ, in a Diels–Alder reaction, with 1,3-butadiene, thus affording adduct (±)-**293**. Epoxidation of olefin (±)-**293**, via bromohydrin formation and base-induced ring closure, followed by methanolysis and lithium aluminium hydride reduction, gave compound (±)-**294** that was itself subjected to formylation, hydrolysis, and acetylation steps to generate the formamide (±)-**295**. Treatment of this last compound with POCl_3_ gave, after methanolysis of the initially formed product, amino-ketal (±)-**296**, the acetate groups of which were cleaved and the more accessible hydroxyl group within the product diol was then converted into the corresponding tosylate. The sulfonamide (±)-**297** so-formed was treated with DBU and the product olefin (±)-**298** heated in aqueous acetic acid to deliver the target alkaloid (±)-haemanthidine [(±)-**69**].

Tsuda used a related strategy, and now involving a late-stage Pictet–Spengler cyclization reaction, to prepare (±)-haemanthamine [(±)-**227**] [72].

(b) BD-Ring Formation via Creation of the C4-N5 Bond (Biomimetic Intramolecular hetero-Michael Addition).

While less extensively deployed than the C6-C6a bond-forming approach to the crinane framework detailed in the preceeding section, the biomimetic approach (see Scheme 1), involving creation of the C4-N5 bond, has provided another effective means of obtaining the title alkaloids.

**Scheme 38:**
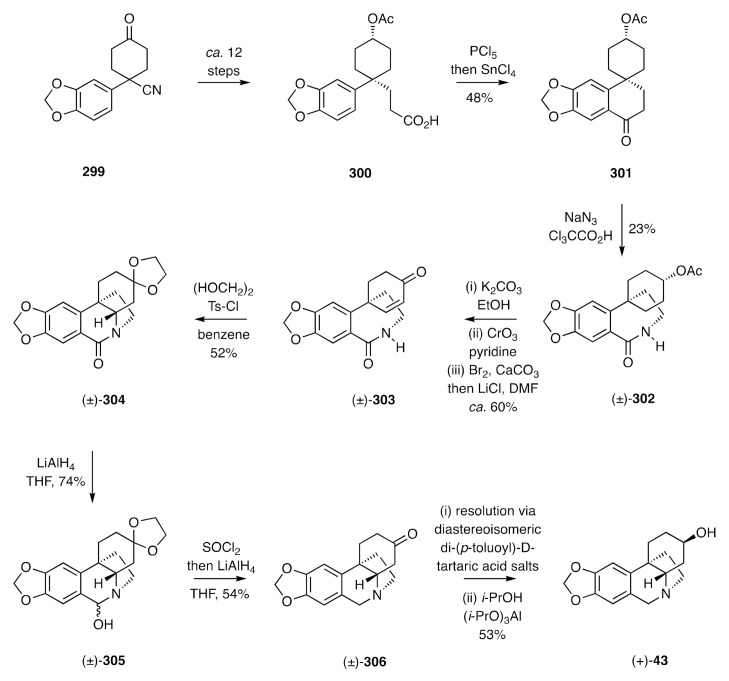
Uyeo’s synthesis of (+)-dihydrovittatine {(+)-elwesine [(+)-**43**]}.

Uyeo was the first to explore this approach, and his efforts culminated in total syntheses of (+)–dihydrovittatine {(+)-elwesine [(+)-**43**]} and related compounds [74,75,76]. Thus, the 4,4-disubstituted cyclohexanone **299** (Scheme 38) was converted over *ca*. twelve steps into the γ-arylated butanoic acid **300**, the acid chloride derivative of which was subjected to an intramolecular Friedel–Crafts acylation reaction to afford the spiro-annulated α-tetralone **301**. Subjection of this last compound to a Schmidt reaction afforded a mixture of lactam (±)-**302 [77,78]** and its regio-isomer with the former being elaborated over three straightforward steps into the enone (±)-**303**. This last compound is “primed” for a biomimetic C4-N5 bond forming event, and the best way to achieve this was under acidic conditions and with ethylene ketal formation following the key cyclization event to afford product (±)-**304**.

A two-step manipulation of the lactam carbonyl delivered, *via* the isolable intermediate (±)-**305**, the ketone (±)-**306**, the constituent enantiomers of which could be resolved using di-(*p*-toluoyl)-d-tartaric acid, and the (+)-form of which could be reduced under Meerwein–Ponndorf-Verley conditions to give (+)-dihydrovittatine {(+)-elwesine [(+)-**43**]}. Analogous reduction of the (−)-form of ketone **306** gave (−)-dihydrocrinine {(−)-elwesine [(−)-**43**]}. 

**Scheme 39:**
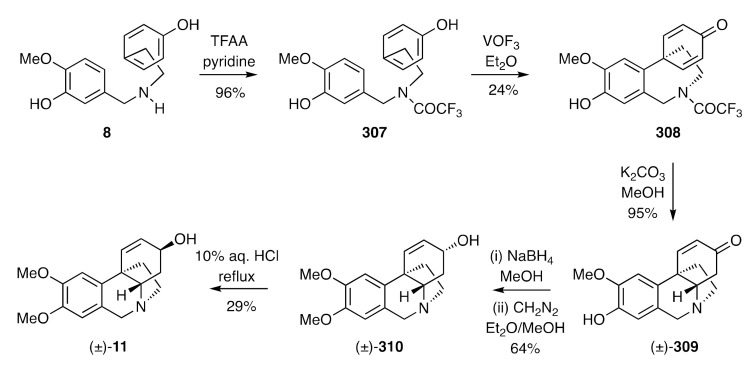
Schwartz’s synthesis of (±)-maritidine [(±)-**11**].

The synthesis of (±)-maritidine [(±)-**11**] reported by Schwartz [79] in 1970 had two biosynthetically relevant features associated with it. The first of these, as shown in Scheme 39, was the intermolecular *para*-*para*-oxidative coupling, using vanadium oxytrifluoride, of the readily available trifluoroacetamide derivative, **307**, of *O*-methylnorbelladiene (**8**) and its conversion, thereby, into the tricyclic product **308**. On treatment of the last compound with potassium carbonate in methanol, the associated amide residue was cleaved, and the resulting free amine engaged in a biomimetic and intramolecular hetero-Michael addition reaction to the pendant cyclohexadienone to form the tetracyclic isomer (±)-**309**. Reduction of this enone with sodium borohydride followed by *O*-methylation of the phenolic group in the product allylic alcohol then gave compound (±)-**310**, the epimer of target [(±)-**11**]. Upon treating compound (±)-**310** with 10% aqueous HCl under reflux, (±)-maritidine [(±)-**11**] was then obtained in 29% yield (with 33% of its precursor also being recovered from the reaction mixture).

**Scheme 40:**
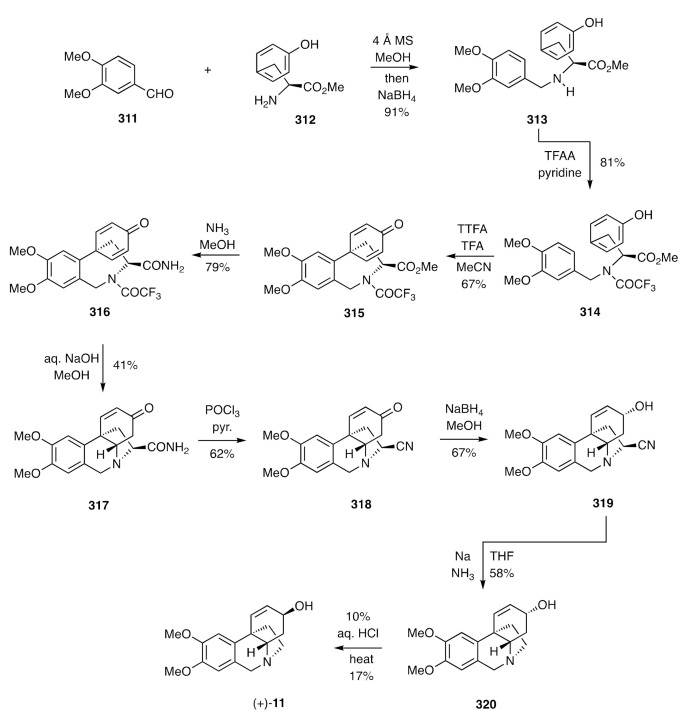
Tomioka’s synthesis of (+)-maritidine [(+)-**11**].

A number of variations on this theme have emerged in the interim, with one of the first being reported by Tomioka (Scheme 40) [80] and allowing for the synthesis of (+)-maritidine [(+)-**11**]. The reaction sequence started with a reductive amination reaction involving veratraldehyde (**311**) and *L*-tyrosine methyl ester (**312**), thus delivering amine **313**. Conversion of this, under standard conditions, into the corresponding trifluoroacetamide **314** provided the substrate for exploring the pivotal oxidative *para*-*para* coupling reaction. After screening a range of reagents for carrying out this conversion, it was established that a solution of thallium trifluoroacetate (TTFA) in TFA was most effective for this purpose and allowed for the formation of the cyclohexadienone **315** in good yield. When this coupling product was treated with ammonia in methanol, the amide **316** was obtained, and on exposing this, in turn, to aqueous sodium hydroxide, then the trifluoroacetamide residue was cleaved, and the liberated amine underwent a completely diastereoselective hetero-Michael addition reaction to generate the tetracyclic compound **317**. The now superfluous carboxamide moiety needed to be excised at this point, and this was achieved by its conversion into the corresponding nitrile **318** and, prior to removal of this, the associated enone unit was subject to 1,2-reduction, thus providing the allylic alcohol **319**. Subjection of this last compound to Birch reduction conditions resulted in removal of the nitrile group and formation of (+)-3-*epi*-martidine [(+)-**320**], while heating this product in aqueous HCl resulted in epimerization, thus providing (+)-maritidine [(+)-**11**] itself, albeit in modest yield.

Since the publication of this work, Kita has shown [81,82] that the conversion **314** → **315** can be achieved in 64% yield using PhI(OCOCF_3_)_2_ (PIFA) in trifluoroethanol at –40 °C and thereby avoiding the need to use highly toxic thallium salts. In a related vein, Ley has used [83], as part of an orchestrated multi-step sequence involving polymer-supported reagents, a hypervalent iodine system to effect oxidative cyclization of the decarboxmethoxy analogue of compound **314**. By such means, operationally simple syntheses of both (±)-oxomaritidine [(±)-**10**] and (±)-3-*epi*-maritidine [(±)-**320**] were realized.

**Figure 8:**
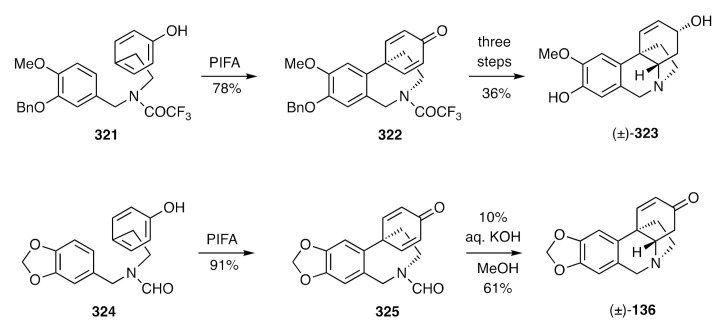
Key aspects of Node’s syntheses of (±)-siculine [(±)-**323**] (**top**) and (±)-oxocrinine [(±)-**136**] (**bottom**).

Node has described [84] related syntheses of (±)-siculine [(±)-**323**] and (±)-oxocrinine [(±)-**136**], the key features of which are shown in Figure 8. Thus, PIFA-mediated oxidative coupling of compound **321** afforded the cyclohexadienone **322**, and the latter compound was readily elaborated to the natural product [(±)-**323**] through debenzylation, base-promoted cyclisation, and 1,2-reduction steps. The methylenedioxyaryl-substituted formamide **324** could be similarly cyclized with PIFA to give product **325**, and on treating this with aqueous potassium hydroxide, both hydrolysis and hetero-Michael addition reactions took place to deliver (±)-oxocrinine [(±)-**136**].

**Scheme 41:**
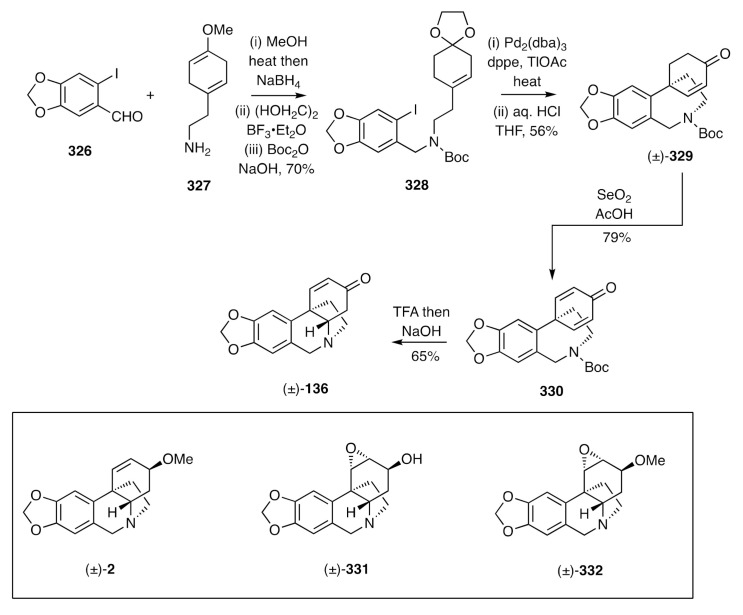
Guillou’s synthesis of (±)-oxocrinine [(±)-**136**] and the structures of (±)-buphanisine [(±)-**2**], (±)-flexinine [(±)-**331**], and (±)-augustine [(±)-**332**] obtained by derivatization thereof.

Guillou used a distinct approach to generate the *N*-Boc protected equivalent of compound **325** and, thereby, (±)-oxocrinine [(±)-**136**] [85]. Thus, as shown in Scheme 41, Schiff base condensation of piperonal **326** with amine **327** followed by reduction of the resulting imine, ethylene ketal formation, and Boc protection of the 2°-amine residue gave the carbamate **328**, which was subjected to an intramolecular Heck reaction. As a result, the spirocyclic product (±)-**329** was obtained and oxidized to the cyclohexadienone **330** using selenium dioxide in acetic acid. Treatment of the latter compound with TFA and the amine salt so-formed with sodium hydroxide resulted in the anticipated hetero-Michael addition reaction and formation of (±)-oxocrinine [(±)-**136**].

Guillou also demonstrated [85] that (±)-oxocrinine [(±)-**136**] could be elaborated by straightforward means to (±)-buphanisine [(±)-**2**], (±)-flexinine [(±)-**331**], and (±)-augustine [(±)-**332**].

**Scheme 42:**
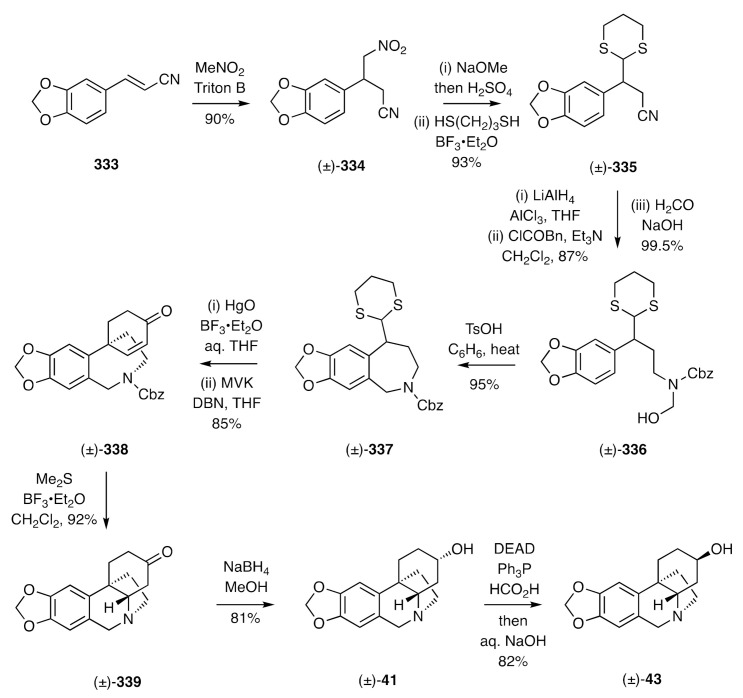
Sánchez’s synthesis of (±)-dihydrooxocrinine [(±)-**339**].

In yet another refinement of Uyeo’s pioneering work (Scheme 38), Sánchez established a synthesis of (±)-dihydrooxocrinine [86] and, thereby, its reduction products (±)-elwesine [(±)-**43**] and (±)3-*epi*-elwesine [(±)-**41**]. In this instance, the approach involved the formation of a hydrobenzoazepine, Robinson-type annulation of which provided a spiro-annulated cyclohexenone residue that could be engaged in a biomimetic and intramolecular hetero-Michael addition reaction to establish the crinane framework. Thus, as shown in Scheme 42, cinnamonitrile **333** was treated with nitromethane in the presence of base to give adduct (±)-**334**, and this was converted, via the corresponding dimethyl acetal, into the dithioacetal (±)-**335**. Reduction of the nitrile residue within the last compound, protection of the resulting amine as a carbamate, and treatment of this with formaldehyde in the presence of base furnished the hydroxymethylated compound (±)-**336**. On treating compound (±)-**336** with TsOH in refluxing benzene, a Tscherniac–Einhorn reaction took place to give the hydrobenzoazepine (±)-**337**. Cleavage of the associated dithioacetal then gave the corresponding aldehyde, which was engaged in a Robinson annulation using methyl vinyl ketone (MVK) and 1,5-diazabicyclo[4.3.0] -non-5-ene (DBN). This gave the spiro-annulated cyclohexenone (±)-**338**. The carbamate moiety associated with this last compound was then cleaved by treatment with dimethyl sulfide in the presence of BF_3_•Et_2_O, and the amine so liberated engaged in an *in situ* hetero-Michael addition reaction to give (±)-dihydrooxocrinine [(±)-**339**]. Reduction of this ketone with sodium borohydride gave (±)-3-*epi*-elwesine [(±)-**41**] exclusively, and upon subjecting this to a Mitsunobu reaction, using formic acid as the nucleophile, and saponifying the product formate (±)-elwesine [(±)-**43**] was obtained. The application of a bromination/dehydrobromination reaction sequence to ketone [(±)-**339**] also allowed for the preparation of (±)-oxocrinine [(±)-**136**].

**Scheme 43:**
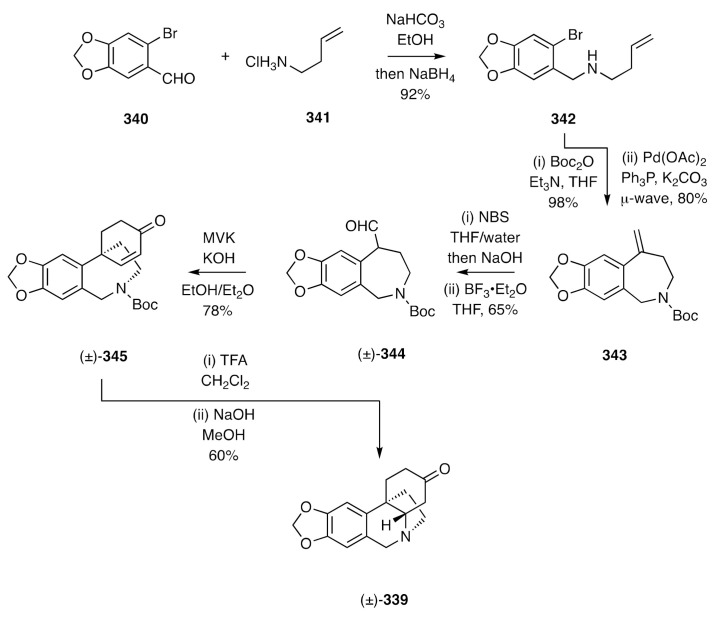
Yang’s modification of Sánchez’s synthesis of (±)-dihydrooxocrinine [(±)-**339**].

Yang has reported [87] a variation of the Sánchez synthesis that has led to (±)-dihydrooxocrinine [(±)-**339**] (Scheme 43). So, reductive amination of bromopiperonal **340** using the hydrochloride salt, **341**, of homoallyl amine gave the 2°-amine **342**, the nitrogen of which was protected with a Boc group. The resulting carbamate was engaged in an intramolecular Heck reaction to afford the hydrobenzoazepine **343**. The associated exocyclic olefin was then converted, via cyclization of the initially formed bromohydrin, into the corresponding spiro-epoxide, which rearranged under Lewis acid conditions to give aldehyde (±)-**344**. This last compound was subjected to a Robinson-type annulation using MVK in the presence of potassium hydroxide, and the cyclohexenone so formed treated, sequentially, with TFA and sodium hydroxide to deliver (±)-dihydrooxocrinine [(±)-**339**].

**Scheme 44:**
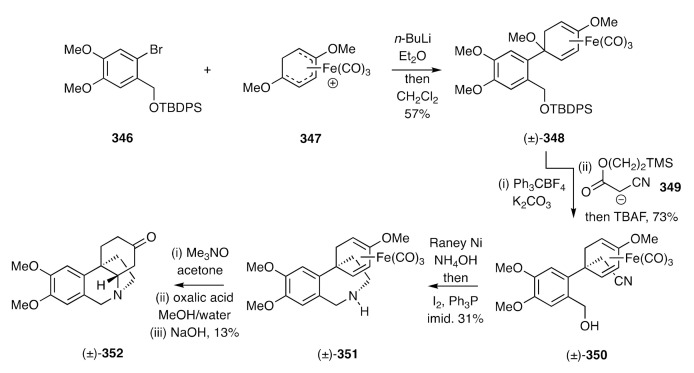
Stephenson’s synthesis of (±)-dihydrooxomaritidine [(±)-**352**].

Stephenson has employed [88,89], as a crucial intermediate, an arylcyclohexadienyliron complex in a biomimetic synthesis of (±)-dihydrooxomaritidine. This starts (Scheme 44) with the reaction of aryl halide **346** with *n*-BuLi and the addition of the ensuing lithio-species to cation **347** to form adduct (±)-**348**. Hydride ion abstraction from the last compound using trityl tetrafluoroborate and reaction of the resulting cation with anion **349** followed by treatment of the adduct so-formed with TBAF then afforded nitrile (±)-**350**, which was itself reduced with Raney nickel in the presence of ammonium hydroxide. The amino-alcohol thereby obtained was treated with Ph_3_P and molecular iodine in the presence of imidazole to afford, via an internal *N*-alkylation process, the spiro-annulated hydrobenzoazepine (±)-**351**. Treatment of this last compound with trimethylamine *N*-oxide resulted in metal decomplexation and mild acid hydrolysis of the liberated enol ether then afforded the corresponding enone that underwent the anticipated biomimetic hetero-Michael addition reaction on treatment with base to give, albeit in modest overall yield, the target (±)-dihydrooxomaritidine [(±)-**352**].

**Scheme 45:**
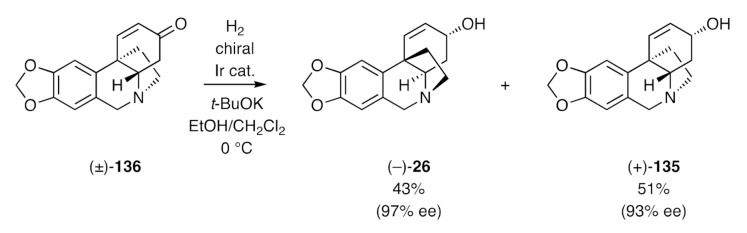
Zhou’s conversion, through asymmetric hydrogenation, of enone (±)-**136** into (−)-crinine [(−)-**26**] and (+)-*epi*-crinine [(+)-**135**].

Zhou has described [90] an asymmetric hydrogenation process, using a homochiral iridium catalyst, that allows for the direct conversion of enones such as, for example, (±)-**136** into (–)-crinine [(–)-**26**] and (+)-*epi*-crinine [(+)-**135**] (Scheme 45), each of which was obtained in good enantiomeric excess. These products were most readily obtained in pure form by conversion into their respective benzoyl esters, chromatographic separation of these, and subsequent independant saponification of each. By employing this approach, an impressive range of crinine alkaloids could be obtained in highly enantiomerically enriched form.

**Scheme 46:**
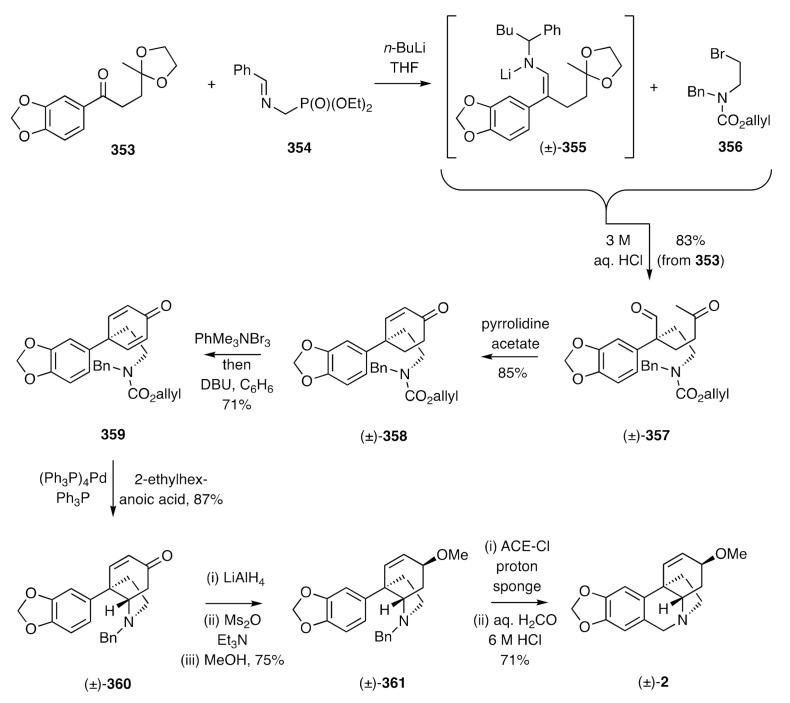
Martin’s synthesis of (±)-buphanisine [(±)-**2**].

Martin has described [91] total syntheses of (±)-crinine [(±)-**26**] and (±)-buphanisine [(±)-**2**] that deploy, as a crucial element, a biomimetic process (although the final ring-forming step involves a Pictet–Spengler reaction). So, as shown in Scheme 46, the readily available 1,4-diketone mono-ketal **353** was treated with phosphonate ester **354** in the presence of *n*-BuLi to afford the three-fold adduct (±)-**355**. This compound was then C-alkylated, in situ, with bromide **356** to yield, after acid treatment, keto-aldehyde (±)-**357**. Upon exposure of the latter compound to pyrrolidine acetate then aldol and dehydration reactions took place to produce cyclohexenone (±)-**358**, which was itself subjected to a bromination/dehydrobromination sequence to afford the cyclohexadienone **359**. Cleavage of the carbamate residue associated with the last compound using Pd[0] revealed the corresponding 2°-amine, which underwent the expected biomimetic hetero-Michael addition reaction to afford the C3a-arylated hydroindole (±)-**360**. This compound was then reduced in a 1,2-fashion with lithium aluminium hydride to give an epimeric mixture of the corresponding allylic alcohols. Mesylation of these alcohols and methanolysis of the resulting esters allowed the ether (±)-**361** to be obtained regio- and stereo-selectively. Hydrogenolysis of the benzylamine moiety within compound (±)-**361** using α-chloroethyl chloroformate (ACE-Cl) in the presence of 1,8-bis(dimethylamino) -naphthalene (proton sponge) followed by heating gave the anticipated 2°-amine that upon subjection to standard Pictet–Spengler conditions delivered (±)-buphanisine [(±)-**2**]. Simple modifications to the closing stages of this reaction sequence also provided access to (±)-crinine.

(c) C-ring Formation via Creation of the C1–C2-Bond (Intramolecular Aldol Condensation)

**Scheme 47:**
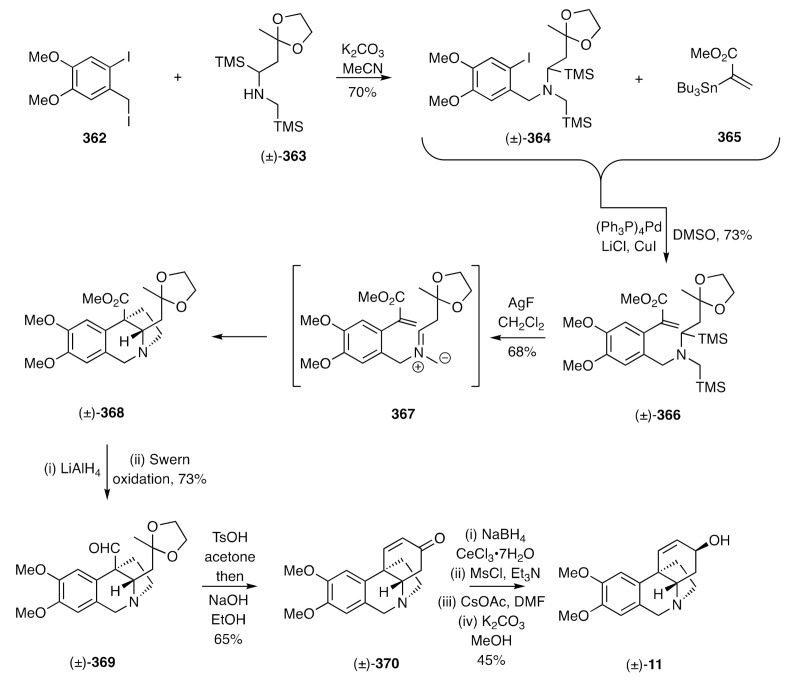
Pandey’s synthesis of (±)-maritidine [(±)-**11**].

In yet another important contribution to the synthesis of crinine alkaloids, Pandey has described [92,93,94] syntheses of (±)-crinine [(±)-**26**] and (±)-maritidine [(±)-**11**] that employ the intramolecular [3+2]cycloaddition of an azomethine ylide to a pendant styrene, thus simultaneously assembling the B- and D-rings of the target. The construction of the C-ring then follows using, in the final ring-forming step, an intramolecular aldol reaction analogous to the conversion (±)-**357** → (±)-**358** used in the Martin synthesis shown immediately above (Scheme 46). The reaction sequence is shown in Scheme 47 and involves, in the opening stages, reaction of the benzyl iodide **362** with the 2°-amine (±)-**363**. Stille-type cross-coupling of the product, (±)-**364**, of this reaction with the stannylated acrylate **365** then gave compound (±)-**366**. Treatment of bis-silane (±)-**366** with silver fluoride resulted in the generation of the pivotal azomethine ylide **367**, which engaged in an intramolecular [3+2] cycloaddition reaction with the pendant acrylate residue, thus affording compound (±)-**368**, the ester residue of which was reduced to the corresponding alcohol, and this was oxidized to the aldehyde (±)-**369**. An acid-catalyzed trans-ketalisation reaction with acetone then followed, and the product keto-aldehyde was treated with base, resulting in C1–C2-bond formation and, thereby, assembly of the C-ring as embodied in enone (±)-**370**. Luche reduction of the last compound, mesylation of the resulting allylic alcohols, and reaction of the esters so formed with caesium acetate gave predominantly a single product that upon reaction with potassium carbonate in methanol afforded (±)-maritidine [(±)-**11**]. An analogous reaction sequence was used to prepare (±)-crinine [(±)-**26**].

(d) C-ring Formation via Creation of the C2–C3-Bond (Carbonyl Ene Reaction)

**Scheme 48:**
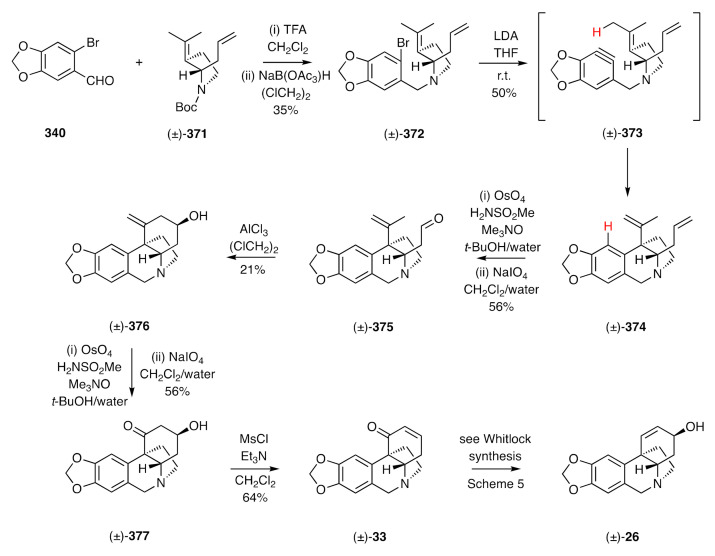
Lauten’s synthesis of (±)-crinine [(±)-**26**].

In another distinctive route to (±)-crinine [(±)-**26**], Lautens detailed [95] an intramolecular aryne ene reaction as a pivotal step and that, as a consequence, set up a second (carbonyl) ene reaction and thus allowing, in the closing stages and through C2–C3-bond formation, assembly of the C-ring. The reaction sequence is shown in Scheme 48 and started with reductive amination of bromopiperonal **340** by the 2°-amine obtained by deprotection of carbamate (±)-**371** with TFA. On treating the ensuing 3°-amine (±)-**372** with LDA, dehydrobromination of the arene took place, and the resulting aryne (±)-**373** underwent the anticipated ene reaction to afford compound (±)-**374** embodying the B- and D-rings of the target. Selective oxidative cleavage of the mono-substitited double bond within diene (±)-**374** afforded aldehyde (±)-**375** that engaged in a Lewis acid-catalyzed carbonyl ene reaction to afford, in a diastereo-selective process, the homoallylic alcohol (±)-**376**. Oxidative cleavage of the double bond within this last compound then gave the β-hydroxy ketone (±)-**377**, which, upon reaction with methanesulfonyl chloride in the presence of triethylamine, afforded enone (±)-**33**, an advanced intermediate associated with Whitlock’s synthesis of (±)-crinine [(±)-**26**].

(e) D-ring Formation via Creation of the N5-C12 Bond (Intramolecular Alkylation at Nitrogen with Accompanying Ethano-bridge Formation).

**Figure 9:**
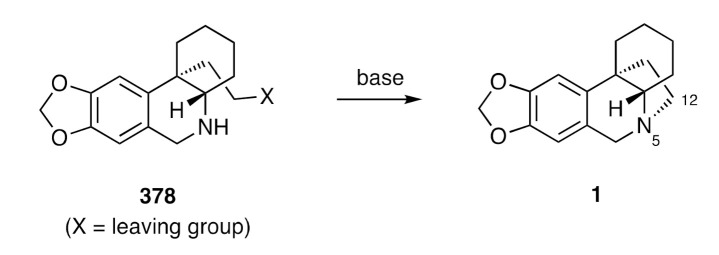
The N5–C12-bond forming process leading to ethano-bridge formation and establishment of the crinane D-ring.

Another effective approach used to assemble the crinane framework **1** involves treating a substrate of the general form **378** (Figure 9) with base and thereby, through alkylation at nitrogen, establishing the N5-C12 bond and the associated D-ring.

**Scheme 49:**
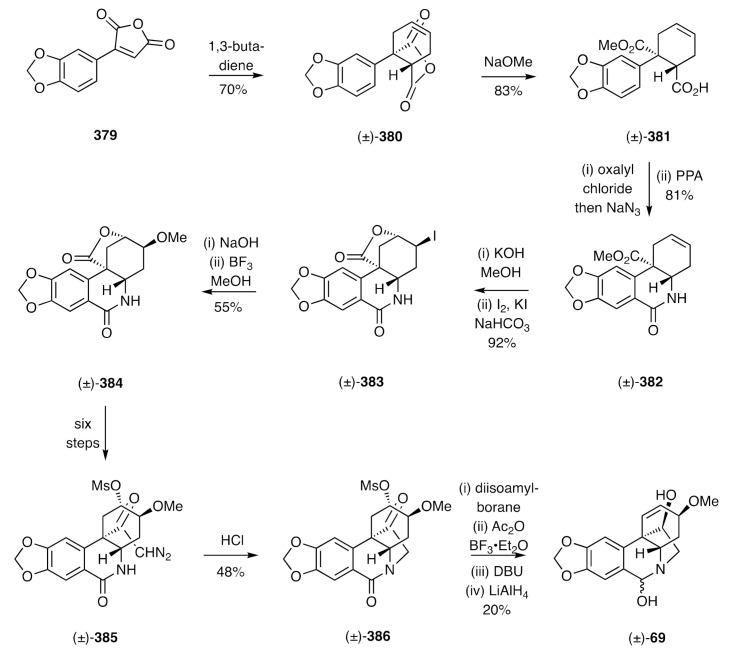
Hendrickson’s synthesis of (±)-haemanthidine [(±)-**69**].

Hendrickson was the first to employ this strategy and used it to great effect in the synthesis of haemanthidine [(±)-**69**] as shown in Scheme 49 [96,97,98]. Thus, the arylated maleic anhydride **379** served as the dienophile in a Diels Alder reaction with 1,3-butadiene, and the adduct (±)-**380** so-formed was treated with sodium methoxide to give the half-ester (±)-**381**. The acid residue associated with the last compound was subjected to a Curtius rearrangement, and the resulting isocyanate cyclized in polyphosphoric acid (PPA) to give lactam (±)-**382**. Saponification of the angular ester residue and engagement of the resulting acid salt in an iodo-lactonisation reaction gave compound (±)-**383**, and treatment of this with sodium hydroxide resulted in epoxide formation. Opening of the three-membered ring using boron trifluoride in methanol then afforded the lactone methyl ether (±)-**384**. A series of conventional manipulations of the lactone residue then followed, including conversion of the liberated alcohol into the mesylate and the acid into the corresponding diazoketone to afford compound (±)-**385**. Treament of this tricyclic intermediate with HCl resulted in N5-C12 bond formation and the creation of the ethano-bridge as manifest in compound (±)-**386**. The ketone carbonyl within this last compound was stereoselectively reduced using diisoamylborane and the resulting alcohol acetylated. Elimination of the elements of methanesulfonic acid from this bis-ester was accomplished using DBU and thereby installing the Δ^1,2^-double bond of the target. The acetate residue was then cleaved and the lactam carbonyl reduced with lithium aluminium hydride to give haemanthidine [(±)-**69**].

**Scheme 50:**
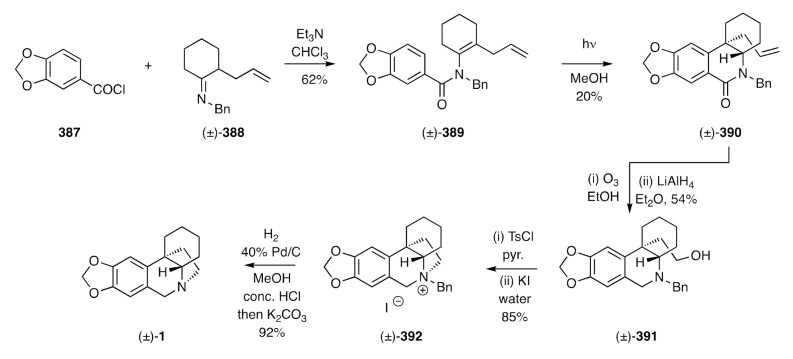
Ninomiya’s synthesis of (±)-crinane [(±)-**1**].

In the only photochemical approach to the crinane framework that seems to have been explored to date, Ninomiya started (Scheme 50) [99,100] by reacting piperonyl chloride **387** with the imine (±)-**388** and then subjecting the product enamide (±)-**389** to a photocyclization reaction to afford lactam (±)-**390**. Ozonolytic cleavage of the allyl group within this product and reduction of the ensuing aldehyde then gave alcohol (±)-**391**, and the derived tolsylate was reacted with potassium iodide in hot water to effect construction of the ethano-bridge and formation of the quaternary ammonium salt (±)-**392**. Exposure of this salt to hydrogen in the presence of palladium on carbon and concentrated HCl then gave, after basic work up, (±)-crinine [(±)-**1**].

**Scheme 51:**
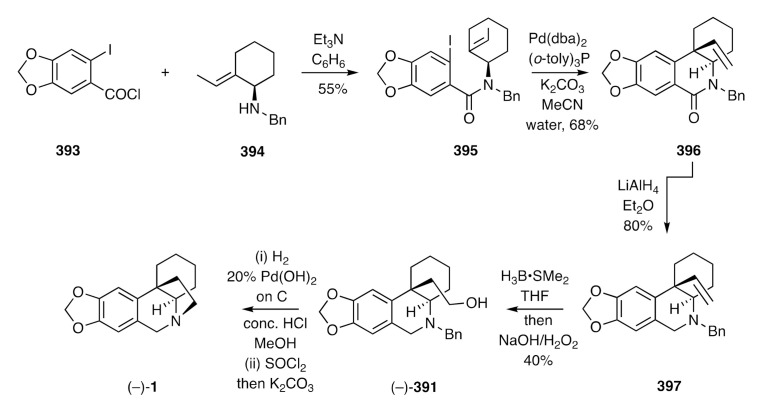
Grigg’s synthesis of (−)-crinane [(−)-**1**].

In Grigg’s synthesis of (–)-crinane [(–)-**1**] (Scheme 51) [101] acid chloride **393** was reacted with the homochiral allylic amine **394** (itself prepared via Overman rearrangement of an allylic alcohol obtained in homochiral form through kinetic resolution of the racemate using Sharpless asymmetric epoxidation techniques) to give amide **395**. Subjection of the last compound to a palladium-catalyzed cyclisation led, in both a regio- and stereo-selective manner, to the lactam **396**, which incorporated the necessary trans-junction between the B- and C-rings of the developing crinane framework. Hydroboration/oxidation of the angular vinyl group associated with compound **396** gave amino-alcohol (−)-**391**, the racemic form of which served as a key intermediate in the Ninomiya synthesis described immediately above (Scheme 50). In the present example, compound (−)-**391** was debenzylated using hydrogen in the presence of Pearlman’s catalyst and the product 2°-amine simply treated with thionyl chloride to effect ring closure and thereby establish the ethano-bridge and completing the synthesis of (−)-crinane [(−)-**1**].

**Scheme 52:**
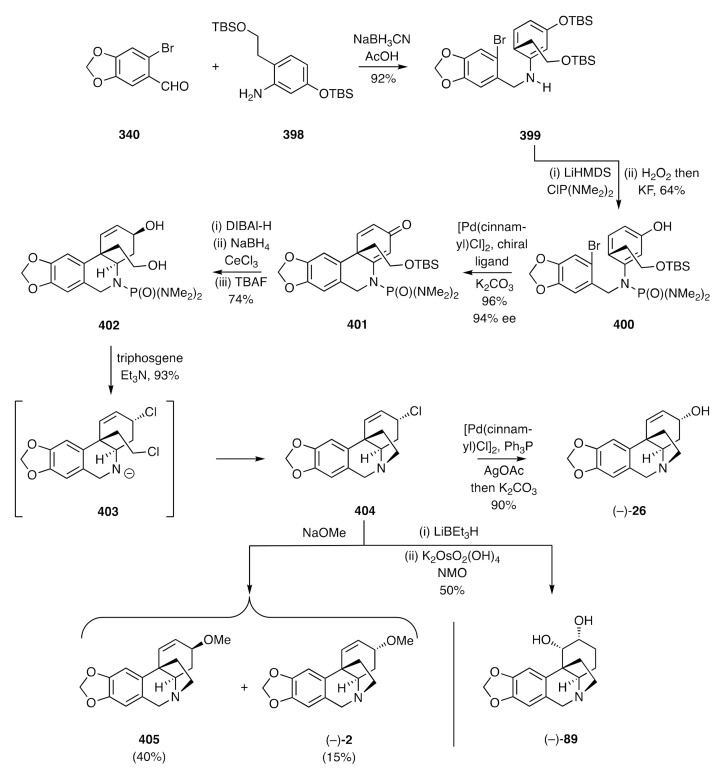
Tang’s syntheses of (−)-crinine [(−)-**26**], (−)-buphanisine [(−)-**2**] and (−)-amabiline [(−)-**89**].

The synthesis of (−)-crinine [(−)-**26**] reported in 2017 by Tang [102,103] employs an intriguing variation of the *para-para*-biaryl coupling that has featured extensively in the biomimetic syntheses of the title alkaloids. The route starts (Scheme 52) with a reductive amination reaction between the bromopiperonal **340** and aniline **398** that produces the 2°-amine **399**. Protecting the amine nitrogen in this product with a bulky phosphoramide group and selective cleavage of the phenolic TBS ether afforded compound **400**, which served as the substrate for a palladium-catalyzed and enantioselective dearomative cyclization, thus delivering the cyclohexadienone **401**. Upon successive treatment of this compound with DIBAl-H, the Luche reagent, and TBAF, the diol **402** was obtained. On exposure of this tricyclic product to triphosgene the associated hydroxyl groups were converted into the corresponding chlorides, and the phosphoramide unit was cleaved. The resulting dichlorinated dialkylamide **403** then cyclized, in situ, and thereby established the ethano-bridge and the full crinane framework as embodied in compound **404**. Treatment of this allylic chloride with silver acetate in the presence of a palladium catalyst followed by treatment of the product acetate with potassium carbonate then gave (−)-crinine [(−)-**26**] stereoselectively and in good yield.

Compound **404** could also be reductively dechlorinated using Superhydride (LiBEt_3_H) and the product cyclohexene then stereoselectively dihydroxylated to give (−)-amabiline [(−)-**89**]. In contrast, reaction of the same allylic chloride with sodium methoxide gave a mixture of (−)-buphanisine [(−)-**2**] and its epimer (−)-**405** with the latter predominating.

### 1.2. As Yet Unexplored Disconnections for Final Ring(s) Formation

**Figure 10:**
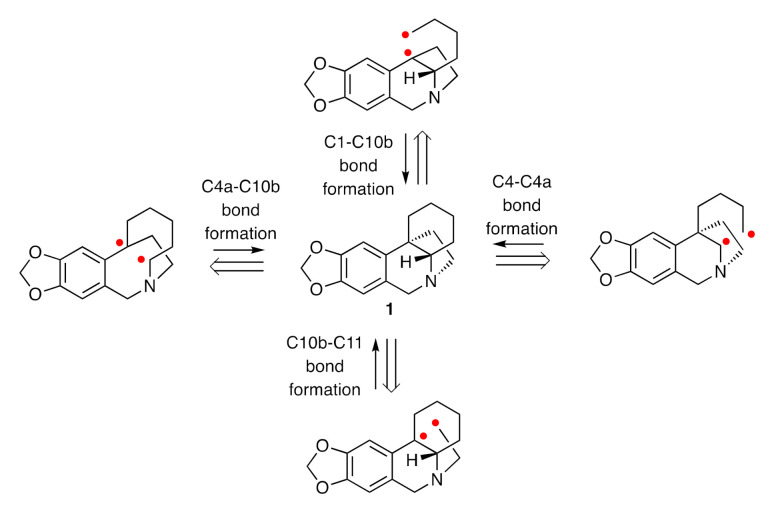
Some possibilities for as yet unexplored final ring-bond forming steps leading to the crinane framework **1** (the atoms needing to be linked are highlighted by red dots—no particular polarties or processes implied).

Consideration of the approaches detailed above for the construction of the crinine alkaloid framework **1** reveals that there is a multitude of other possible means for assembling this important ring system. By simply confining such possibilities to single and final ring-bond forming events, those shown in Figure 10 illustrate some further options for molecular assembly. Whether such approaches are truly useful remains to be seen and will depend, in large part, on how readily the relevant precursors can be assembled and whether or not such approaches lend themselves to asymmetric synthesis and/or the formation of novel and otherwise less accessible derivatives.

Of course, multiple bond forming and domino processes, which remain less explored approaches, are also likely, by virtue of their inherent efficiencies, to provide fertile ground for future research.

## 2. Conclusions

The more than sixty-year history of the development of synthetic approaches to and total syntheses of the title alkaloids parallels the evolution of organic chemistry more generally. Indeed, it might be argued that the story of crinine syntheses over this extended period provides something of a time capsule that allows the viewer to “observe” the changing research priorities and techniques that have come into play during this time. Ironically, perhaps, while the target structures have remained constant, that is not the case with laboratory approaches to them. In the earlier phases of such work, the priority was the target. More recently, however, the crinines have become vehicles for showcasing the value of new methodologies. Precisely how this topic might evolve over the next few decades is difficult to predict and may well be influenced by the identification, if any, of crinines with real clinical value [104]. Given the capacity of crinines to serve as precursors to other ring systems, including those associated with various natural products [6,7,105,106,107,108], new uses of the title compounds are likely to emerge and provide yet further impetus for establishing distinctive routes to them.
